# Activation of HERV-K Env protein is essential for tumorigenesis and metastasis of breast cancer cells

**DOI:** 10.18632/oncotarget.11455

**Published:** 2016-08-20

**Authors:** Fuling Zhou, Ming Li, Yongchang Wei, Kevin Lin, Yue Lu, Jianjun Shen, Gary L. Johanning, Feng Wang-Johanning

**Affiliations:** ^1^ Viral Oncology Program, Center for Cancer and Metabolism, SRI International, Menlo Park, California, USA; ^2^ Department of Clinical Hematology, Zhongnan Hospital, Wuhan University, Wuhan, Hubei, China; ^3^ Department of Radiation and Medical Oncology, Zhongnan Hospital, Wuhan University, Wuhan, Hubei, China; ^4^ Department of Epigenetics and Molecular Carcinogenesis, Science Park, The University of Texas MD Anderson Cancer Center, Smithville, Texas, USA

**Keywords:** HERV-K, breast cancer, shRNA, Ras signaling, tumorigenesis and metastasis

## Abstract

Human endogenous retrovirus type K (HERV-K) Env protein was previously demonstrated to be overexpressed in human breast cancer (BC) cells and tissues. However, the molecular pathways driving the specific alterations are unknown. We now show that knockdown of its expression with an shRNA (shRNAenv) blocked BC cell proliferation, migration, and invasion. shRNAenv transduction also attenuated the ability of BC cells to form tumors, and notably prevented metastasis. Mechanistically, downregulation of HERV-K blocked expression of tumor-associated genes that included Ras, p-RSK, and p-ERK. The major upstream regulators influenced by HERV-K knockdown were p53, TGF- β1, and MYC. Of interest, when the HERV-K *env* gene was overexpressed in shRNAenv-transduced BC cells using an HERV-K *env* expression vector, *Ras/Raf/MEK/ERK* pathway signaling was restored. CDK5, which alters p53 phosphorylation in some cancers, was upregulated and p53 was downregulated when HERV-K was overexpressed. CDK5 is also a mediator of TGF-β1-induced epithelial-mesenchymal transition and migration in cancer cells, and is involved in tumor formation. Importantly, reductions in migration, invasion, and transformation of BC cells stably transduced with shRNAenv was reversed after adding back a vector with a synonymous mutation of HERV-K *env*. Taken together, these results indicate that HERV-K Env protein plays an important role in tumorigenesis and metastasis of BC.

## INTRODUCTION

Early detection, diagnosis, and treatment of solid tumors that include breast cancer (BC), and particularly metastatic BC, is still a challenge today. The existence of previously unrecognized causative agents of BC may be one reason why a cure for BC has been so elusive. Furthermore, treatment of patients with BC or other cancers has been challenging due to the heterogeneity of the disease and the absence of well-defined molecular targets. One promising etiological factor in BC may be the involvement of an endogenous retrovirus (ERV).

ERV sequences make up > 8% of the human genome [1] and some may show retroviral activity in humans that include tumor induction. Our laboratory showed that the human endogenous retrovirus type K (HERV-K) family, which is usually silenced, commonly becomes induced and activated in BC cells and tissues [2–7]. We confirmed overexpression of HERV-K in BC patients, and the expression significantly increased with disease stage, grade, p53 mutation and lymph node metastasis [6]. There was also significantly increased expression of HERV-K mRNA in sera of BC patients whose cancer had metastasized three years after baseline blood draws, compared with BC patients whose cancer did not metastasize, irrespective of baseline receptor status [4]. Importantly, we did not detect expression of HERV-K in a wide panel of normal human tissues [8, 9]. In further support of a role of HERV-K in metastatic cancers, we observed that HERV-K specific T cells were cytotoxic [5, 10], and HERV-K antibody had anti-tumor effects [6].

In this study we examined whether tumor-promoting activity of the HERV-K Env protein in BC cell lines was mediated by the *Ras/Raf/MEK/ERK* signaling pathway both *in vitro* and *in vivo*.

## RESULTS

### Reduced expression of HERV-K *env* RNA or protein in cell lines transduced with an shRNA targeting HERV-K *env*

The overexpression of HERV-K in various breast cancer cell lines has been demonstrated previously [5, 6, 8]. Higher percentages of HERV-K Env protein expression were demonstrated in BC cell lines (Hs578T, MDA-MB-231, MCF-7, MDA-IBC-3, and BT549) than in HEK293 or HEK293T cells by flow cytometry (FACS), cell enzyme-linked immunosorbent assay (ELISA), immunofluorescence staining (IFS), immunohistochemistry (IHC), and immunoblot (data not shown). siRNA has been used to inhibit the expression of specific messenger RNAs to produce gene silencing effects for cancer therapeutics [11]. Six siRNAs targeting HERV-K *env* were designed and were found to knock down HERV-K expression in 3 BC cell lines (Figure S1A). Furthermore, several siRNAs inhibited MDA-MB-231 BC cell proliferation (Figure S1B), and when either 6 or 12 siRNAs were combined, there was very strong and significant inhibition of HERV-K expression in T47D, SKBR3, and MDA-MB-231 BC cells (Figure S1C). Since shRNA has a lower rate of degradation and turnover relative to siRNA, siRNA 670 was selected and used for synthesis of shRNA (shRNAenv). A scrambled siRNA 670 was used to synthesize shRNAc.

**Figure 1:**
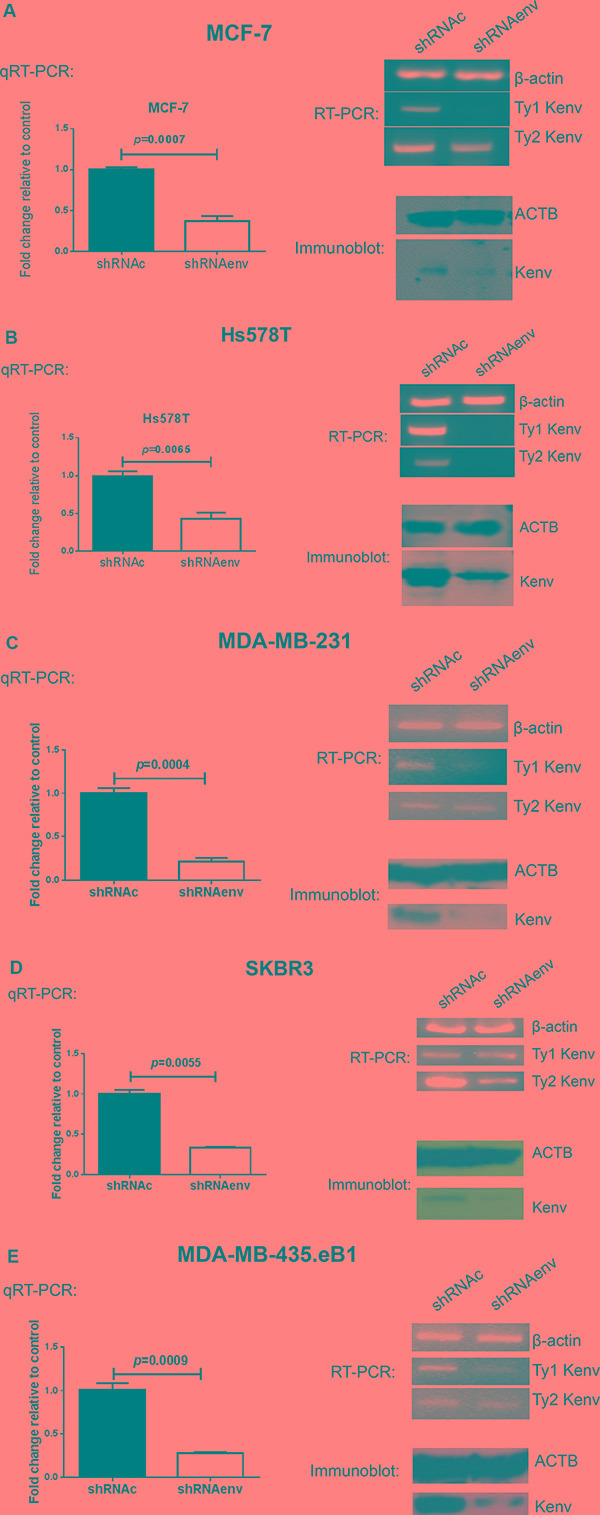
Expression of HERV-K in BC cell lines transduced with shRNAenv vs. shRNAc Quantitative RT-PCR, RT-PCR, and immunoblot using HERV-K specific primers and anti-HERV-K antibody were used to determine whether HERV-K knockdown blocked its expression. Changes in HERV-K expression in shRNAenv relative to controls (shRNAc) were observed by qRT-PCR assays in MCF-7 (**A**), Hs578T (**B**), MDAMB-231 (**C**), SKBR3 (**D**), and MDA-MB-435.eB1 (**E**) cells. The deviation (error bars) represents standard error of the mean (SEM), and the statistical test performed was unpaired *t* test (*n* = 3). Downregulated expression of HERV-K env RNA (type 1) in MCF-7, Hs578T, MDAMB-231, MDA-MB-435.eB1, or type 2 in Hs578T and SKBR3 (type 2 only) cells was demonstrated by RT-PCR. There are two types of HERV-K: type 1 has a 292 bp deletion near the 5′ end of the env gene, whereas type 2 does not have this deletion. RT-PCR is employed to detect the two types of HERV-K env, using primers specific for type 1 or type 2. Downregulated expression of HERV-K Env protein was demonstrated by immunoblot in cell lines transduced with shRNAenv, using an anti-HERV-K monoclonal antibody (6H5). ACTB antibody was used as control.

Transduction with an shRNA targeting HERV-K using a pGreenPuro (System Biosciences, Palo Alto, CA) expression vector (shRNAenv; Figure S2A and S2B) [8], relative to transduction with a scrambled control (shRNAc), led to significantly reduced expression of RNA in MCF-7 (*p* = 0.0007; Figure 1A), Hs578T (*p* = 0.0065; Figure 1B), MDA-MB-231(*p* = 0.0004; Figure 1C), SKBR3 (*p* = 0.0055; Figure 1D), and MDA-MB-435.eB1 (*p* = 0.0009; Figure 1E) cells, as assessed by qRT-PCR using primers described previously [3]. shRNAenv treatment led to reduced expression of HERV-K type 1 in most of the BC cells we tested, and of type 2 in Hs578T and SKBR3 cells, by RT-PCR using primers described previously [2]. Reduced expression of HERV-K Env protein was also detected in the above cell lines by immunoblot (Figure 1A–1E) using the previously described anti-HERV-K monoclonal antibody 6H5 [5, 12].

### Inhibition of cell proliferation, colony formation and cell transformation in shRNAenv transduced cancer cells

**Figure 2:**
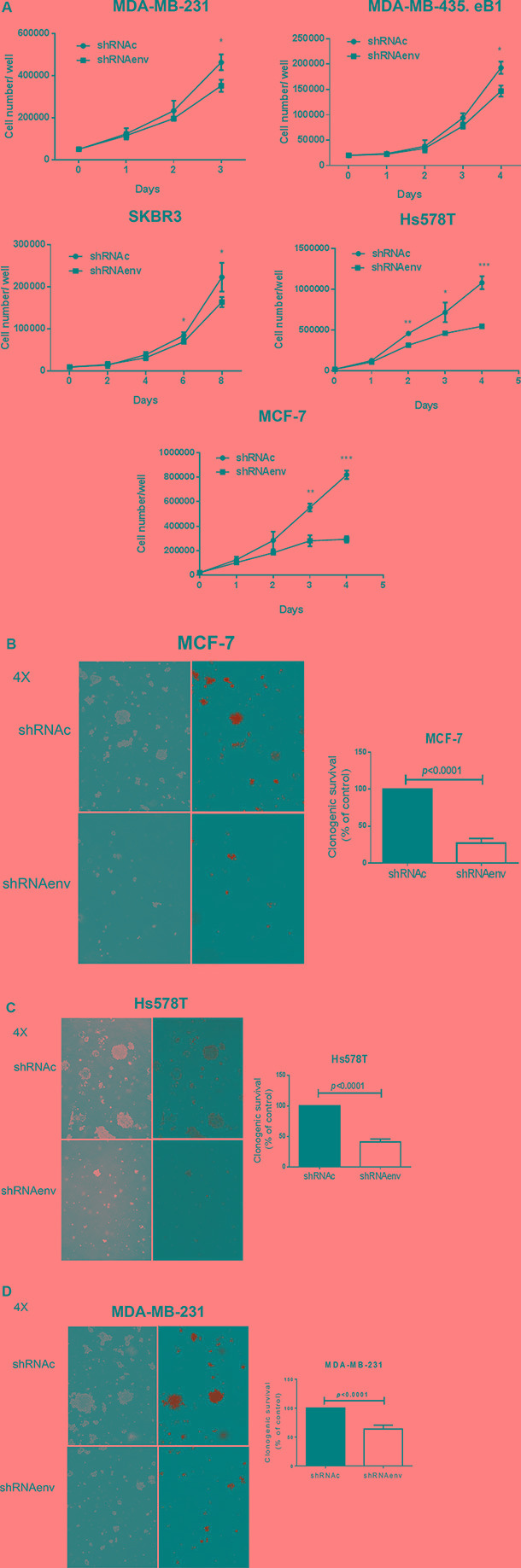
Effect of HERV-K knockdown on cell growth and anchorage-independent growth of BC cells (**A**) Significantly reduced cell proliferation, assessed by counting live cell numbers, was observed for MDAMB-231, MDA-MB-435.eB1, SKBR3, Hs578T, and MCF-7 cells (*n* = 3; unpaired *t* test), after HERV-K knockdown by shRNAenv. An anchorage-independent growth assay was used to determine cell transformation after transduction with shRNAenv and shRNAc (*n* = 9; unpaired *t* test). Reduced colony numbers were demonstrated for MCF-7 (**B**) (*p* < 0.0001; 10 days post-seeding), Hs578T (**C**) (*p* < 0.0001; 10 days post-seeding), MDA-MB-231 (**D**) (*p* < 0.0001; 10 days post-seeding and *p* = 0.0034; 2 weeks post-seeding).

Cell number counts or a proliferation assay [6] showed significantly reduced cell proliferation of MDA-MB-231, MDA-MB-435.eB1, MCF-7, and Hs578T cells on days 3–4, except for SKBR3 cells, which showed decreased proliferation on days 6–8 after shRNA knockdown of HERV-K (Figure 2A). An anchorage-independent growth assay revealed significantly reduced colony formation in MCF-7 (*p* < 0.0001; Figure 2B), Hs578T (*p* < 0.0001; Figure 2C), MDA-MB-231 (*p* < 0.0001 or *p* = 0.0034; Figure 2D), SKBR3 (*p* = 0.0039, Figure S2C), and MDA-MB-435.eB1 (*p* = 0.0046; Figure S2D) cells, after shRNA knockdown of HERV-K. In addition, cell migration, as determined by a scratch assay, was decreased in MDA-MB-231, SKBR3, and MDA-MB-435.eB1 cells transduced with shRNAenv (data not shown). Transwell plate assays also showed reduced migration of MDA-MB-435.eB1 and SKBR3 cells, and reduced invasion of MDA-MB-435.eB1 cells after shRNAenv knockdown of HERV-K (Figure S2E).

### Reduced tumor formation and metastasis to lung *in vivo*

**Figure 3:**
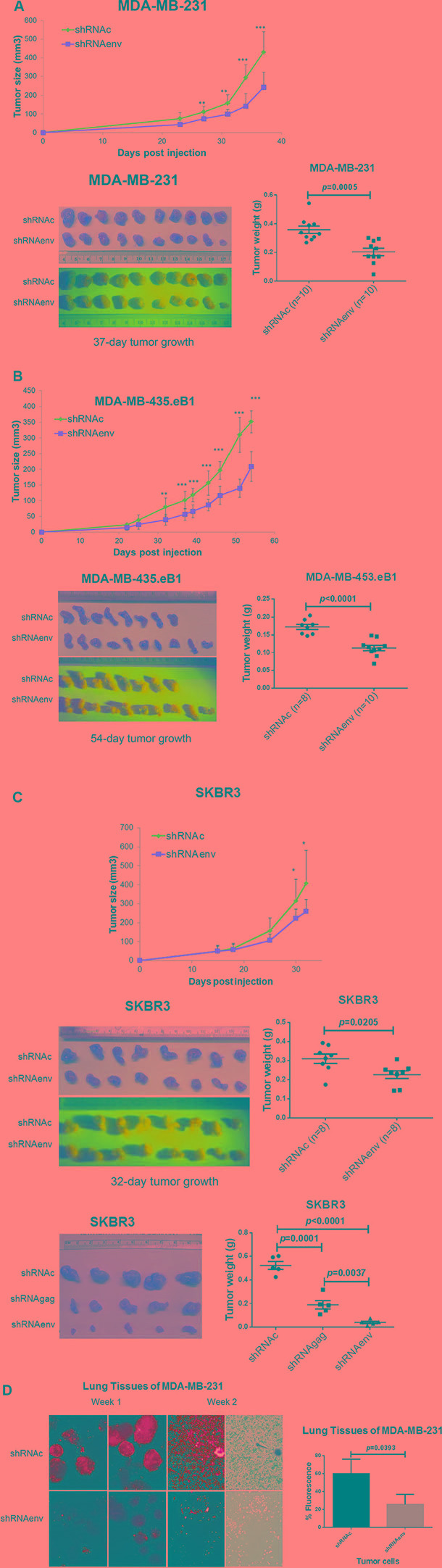
Effect of HERV-K knockdown on tumor growth in mouse xenografts Significantly reduced tumor growth was observed in immunodeficient mice xenografted with MDA-MB-231 (**A**, top), MDA-MB-435.eB1 (**B**, top), and SKBR3 (**C**, top) cells transduced with shRNAenv, compared to cells transduced with shRNAc. Significantly reduced tumor sizes and weights were further demonstrated in these three BC xenografts of MDA-MB-231 (**A**, bottom right), MDA-MB-435.eB1 (**B**, bottom right), and SKBR3 (**C**, middle right) cells (*n* = 8–10; unpaired *t* test). Significantly reduced tumor sizes and weights were also demonstrated for xenografts bearing SKBR3 cells transduced with shRNAenv (**3C**, bottom right) or shRNAgag (*n* = 5; unpaired *t* test). The blue panels show green fluorescence of tumors containing human cells, since BC cells were transfected with shRNAc or shRNAenv expression vectors, which contain a green fluorescent protein (GFP) tag. Tumor sizes and weights were lower in the shRNAenv knockdown mice than in the shRNAgag knockdown mice. Furthermore, reduced metastasis to lung was demonstrated in MDA-MB-231 shRNAenv xenografts compared with shRNAc (**D**; Week 1 and 2). Significantly higher percentages of fluorescent cells were observed in lungs of shRNAc vs, shRNAenv (Week 1; *p* = 0.0393; *n* = 3; unpaired *t* test). Green fluorescent cells indicate human MDA-MB-231 cells.

Significantly reduced tumor growth was observed in mouse xenografts of MDA-MB-231 (Figure 3A), MDA-MB-435.eB1 (Figure 3B), and SKBR3 (Figure 3C) cells transduced with shRNAenv compared with shRNAc. Furthermore, tumor sizes and weights were significantly reduced in mouse xenografts bearing MDA-MB-231 (*p* = 0.0005; Figure 3A), MDA-MB-435.eB1 (*p* < 0.0001; Figure 3B) and SKBR3 (*p* = 0.0205 and *p* < 0.0001; Figure 3C) cell lines transduced with shRNAenv. Reduced tumor weight was also observed in SKBR3 cells transduced with an shRNA targeting HERV-K *gag* (shRNAgag; *p* = 0.0001; Figure 3C), but not as much as in cells transduced with shRNAenv (*p* = 0.0037). Importantly, significantly reduced metastasis to lung (lower green fluorescence in tissue pieces) was demonstrated in mice bearing MDA-MB-231 shRNAenv xenografts compared with those bearing MDA-MB-231 shRNAc xenografts (*p* = 0.0393; Figure 3D). HERV-K *env* RNA expression was significantly downregulated in xenograft tumors of shRNAenv compared with shRNAc MDA-MB-231 (*p* = 0.0449 or *p* = 0.0004; Figure S3A) and MDA-MB-435.eB1 (*p* = 0.0006; Figure S3B) cells by qRT-PCR. These data indicate that reduced tumor sizes may be due to downregulated expression of HERV-K by shRNA.

### Phenotypic changes of BC cells after downregulated expression of HERV-K *env* RNA

**Figure 4:**
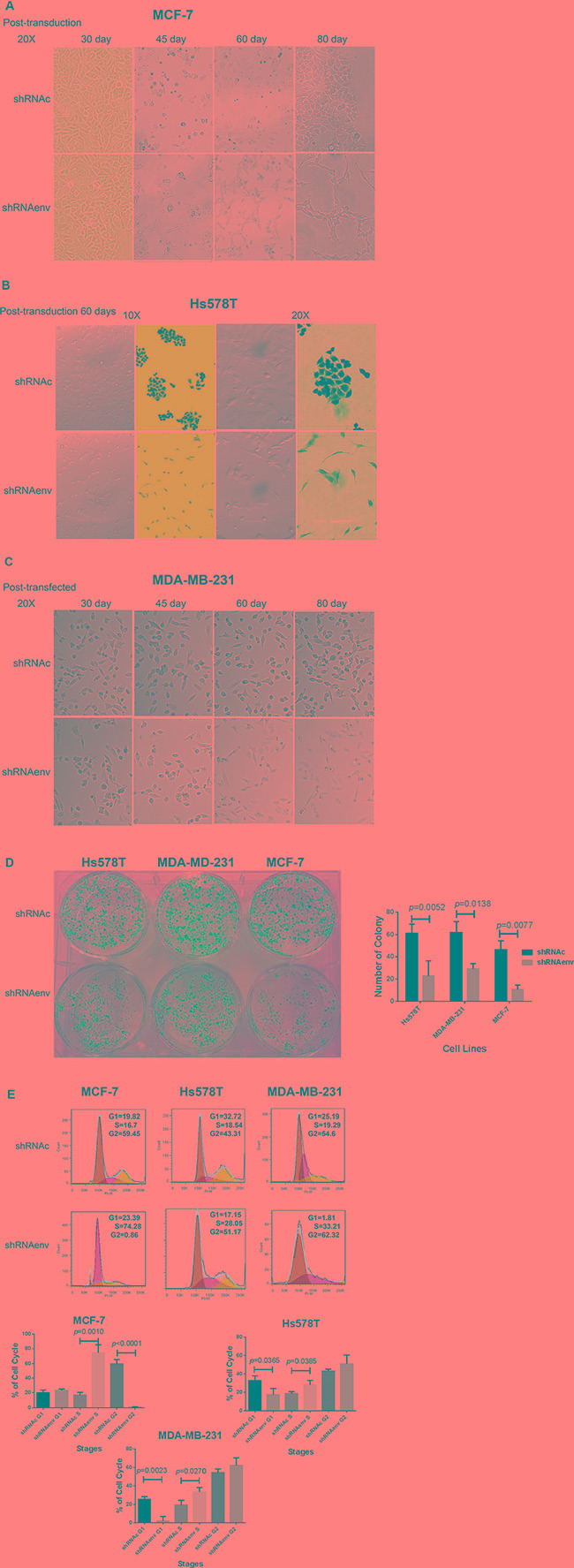
Phenotype changes in BC cells transduced with shRNAenv Changes in phenotype in MCF-7 (**A**), Hs578T (**B**), and MDA-MB-231 (**C**) cells after HERV-K knockdown. Overall, significantly fewer and smaller sized colonies were observed in the three BC cell lines transduced with shRNAenv compared with shRNAc (**D**) Hs578T: *p* = 0.0052; MDA-MB-231: *p* = 0.0138; and MCF-7: *p* = 0.0077; *n* = 4; unpaired *t* test) in the anchorage-independent colony formation assay. Furthermore, FACS analysis revealed significantly increased S phase arrest in the three cell lines after stable transfection with shRNAenv (**E**) *n* = 3; unpaired *t* test). Significantly decreased G1 phase arrest was observed in the two TNBC cell lines but not in the MCF-7 cells. A nonsignificant G2 phase arrest was observed in the two TNBC cell lines.

We observed that long-term culture of BC cells transduced with shRNAenv led to slower cell growth rates, which was accompanied by phenotypic changes in MCF-7 cells at day 45 post-transduction (Figure 4A), and in Hs578T at day 60 post-transduction (Figure 4B). The cells transduced with shRNAenv showed a spindle-like elongated phenotype, compared to cells transduced with shRNAc. However, no significant change in phenotype was noted in MDA-MB-231 cells transduced with shRNAenv after similar numbers of days post-transduction (day 30 to day 80; Figure 4C). An image of cells cultured under anchorage-independent growth conditions from the 3 BC cell lines transduced with shRNAenv or shRNAc is shown in Figure 4D. Significantly reduced numbers of colonies and smaller colony sizes were observed in the 3 BC cell lines transduced with shRNAenv compared with shRNAc cells.

Cell cycle analysis revealed S phase arrest in MCF-7, Hs578T, and MDA-MB-231cell lines transduced with shRNAenv (Figure 4E), compared to these cells transduced with shRNAc or parent cells (parent cell data not shown). Downregulated expression of HERV-K *env* gene caused a significant increase of cells in the S phase of all three BC cell lines and decrease in G1 in two triple negative BC(TNBC) cell lines (Hs578T and MDA-MB-231). There was a corresponding significant decrease of cells in the G_2_-M phases in MCF-7, and a slight but nonsignificant increase of cells in G_2_-M phases in Hs578T and MDA-MB-231 cells. The percentages of cells in various phases are shown in Figure S3C.

### RNA-seq analysis

Knockdown of HERV-K resulted in dramatic decreases in BC cell proliferation *in vitro* and tumor growth *in vivo*, and greatly decreased metastasis to the lung. To address mechanisms underlying HERV-K effects on cell proliferation and metastasis, RNA-Seq was employed to evaluate gene expression in shRNAenv cells compared with shRNAc cells. We then input genes differentially expressed between the shRNAenv and shRNAc, along with their expression levels, into the Ingenuity Pathway Analysis (IPA) program to identify functional networks and signaling pathways that are affected by HERV-K *env* downregulation. Several signaling pathways were identified that had statistically significant *P* values, and multiple biological processes were affected. A summary of Upstream Regulator and Disease or Functions Annotation from these cell lines is shown in Table 1 and Table 2, respectively.

**Table 1:** Ingenuity Pathways Analysis (IPA) of upstream regulators of differentially expressed genes between BC cell lines with and without knockdown of HERV-K env RNA

	MDAMB231				MCF-7		SKBR3				MDAMB435.eB1	
	Upstream Regulator	p-value of overlap	Mechanistic Network	Upstream Regulator	p-value of overlap	Mechanistic Network	Upstream Regulator	p-value of overlap	Mechanistic Network	Upstream Regulator	p-value of overlap	Mechanistic Network
1	TP53	1.72E-42	677 (22)	TP53	6.59E-40	376 (20)	TP53	3.95E-22	189 (16)	TP53	3.30E-11	234 (20)
	MYC	8.79E-31	703 (21)	MYC	4.32E-14	446 (21)	MYC	1.22E-17	205 (17)	MYC	6.87E-11	219 (17)
	HNF4A	3.96E-20	669 (17)	NUPR1	9.74E-19					MYCN	4.13E-14	
	HIF1A	1.15E-16	631 (20)	CCND1	2.76E-15	351 (22)	HIF1A	2.25E-29	207 (18)	SREBF2	3.59E-10	183 (16)
				E2F4	3.01E-15		JUN	9.73E-20	220 (19)	ATF4	8.92E-10	
				RB1	3.40E-15	343 (18)				MITF	4.49E-09	142 (5)
										SMARCA4	1.76E-08	202 (15)
				CDKN2A	1.01E-14	336 (18)						
				E2F1	8.47E-14	334 (16)						
				SMARCB1	2.20E-13	444 (22)						
2	TGFB1	1.06E-40	745 (20)	TGFB1	4.59E-26	494 (22)	TGFB1	8.76E-37	277 (18)	TGFB1	1.96E-20	280 (18)
	HGF	7.70E-18	590 (16)	HGF	2.66E-09	398 (24)	HGF	3.96E-20	224 (17)	HGF	1.67E-08	194 (16)
				IGF2	1.48E-13	379 (17)				TGFB3	1.05E-08	197 (15)
	EGF	1.08E-17	741 (21)	EGF	8.77E-07	347 (23)	EGF	1.67E-19	220 (19)			
							AGT	1.21E-17	184 (17)			
							FGF2	1.35E-23	218 (16)			
3	beta-estradiol	8.26E-36	743 (23)	beta-estradiol	5.09E-27	510 (22)	beta-estradiol	5.01E-27	267 (17)	beta-estradiol	2.95E-11	316 (22)
	hydrogen peroxide	5.57E-19	621 (19)	hydrogen peroxide	2.30E-08	434 (21)				hydrogen peroxide	1.27E-11	206 (17)
	D-glucose	9.84E-17	536 (18)							dihydrotestosterone	2.00E-08	230 (19)
	tretinoin	3.86E-16	767 (22)	tretinoin	1.52 E-12	469 (19)	tretinoin	1.37E-23	279 (18)			
4	TNF	1.98E-29	704 (19)	TNF	5.26E-15	356 (17)	TNF	5.26E-28	262 (18)			
	IFNG	9.02E-22	464 (14)	IFNG	3.38E-14	462 (17)	IL1B	7.22E-21	246 (17)			
	IL1B	5.60E-18	484 (15)	OSM	5.18E-13	434 (23)	OSM	2.63E-20	229 (20)			
				fulvestrant	3.20E-16	399 (21)	salirasib	6.08E-23	215 (15)			
				trichostatin A	4.18E-16	452 (23)	lipopolysaccharide	2.95E-20	254 (17)	sirolimus	1.62E-10	248 (20)
5	dexamethasone	1.05E-28	711 (19)	dexamethasone	1.42E-15	475 (21)	dexamethasone	3.52E-20	275 (19)	5-fluorouracil	1.68E-08	
	lipopolysaccharide	5.70E-24	546 (14)	cisplatin	2.04E-13	467 (20)	deferoxamine	8.81E-20	199 (16)			
	cisplatin	7.44E-17	654 (17)	valproic acid	3.77E-13	449 (21)	actinomycin D	5.39E-18	227 (20)			
	methylprednisolone	2.00E-21	519 (17)	methylselenic acid	4.86E-17	471 (22)						
6	ERBB2	1.05E-24	636 (20)	ERBB2	5.82E-16	344 (22)	ERBB2	2.66E-19	228 (21)	ERBB2	1.95E-12	199 (16)
							SRC	4.10E-18	197 (17)	RAF1	2.36E-11	238 (19)
										PRKCA	6.52E-09	238 (21)
7	KRAS	3.14E-12	655 (25)	KRAS	4.91E-19	428 (19)	KRAS	8.38E-10	211 (19)	KRAS	1.26E-06	236 (17)
	HRAS	1.15E-18	682 (25)	HRAS	3.14E-11	470 (24)	HRAS	7.48E-15	209 (19)	HRAS	1.19E-10	193 (16)
	MGEA5	1.82E-17										
	FN1	2.36E-16	590 (14)							MGEA5	5.75E-10	
8	SYVN1	1.58E-21		PGR	5.55E-14	386 (21)				POR	2.45E-09	
	FSH	1.40E-18	691 (18)	ESR1	1.12E-13	402 (24)				SYVN1	2.58E-09	
	ERK	1.98E-16	539 (18)				PDGF BB	8.63E-21	256 (19)	PDGF BB	7.51E-09	206 (20)
							estrogen receptor	6.30E-24	212 (18)	EGFR	3.71E-08	248 (18)
							U0126	4.03E-21	234 (18)	U0126	1.10E-08	235 (17)
							PD98059	4.85E-28	221(18)			

1 transcription regulator

2 growth factor

3 chemical - endogenous mammalian

4 cytokine

5 chemical drug

6 kinase

7 enzyme

8 Others including transporter, complex, group, and chemical - kinase inhibitor

**Table 2:** Ingenuity Pathways Analysis (IPA) functional annotation of differentially expressed genes between BC cell lines with and without knockdown of HERV-K env RNA

	MDAMB231			MCF-7			SKBR3			MDA-MB-435		
	Functional Annotation	p-Value	# Molecules	Functional Annotation	p-Value	# Molecules	Functional Annotation	p-Value	# Molecules	Functional Annotation	p-Value	# Molecules
1	proliferation of cells	6.47E-32	687	proliferation of cells	8.53E-25	431	proliferation of cells	1.40E-29	236	proliferation of cells	2.29E-11	242
2	apoptosis	7.17E-28	520	apoptosis	5.33E-19	319	apoptosis	2.00E-26	193	necrosis	4.87E-09	177
	necrosis	6.53E-26	506	necrosis	1.84E-18	313	necrosis	2.56E-27	187	apoptosis	5.05E-09	184
	cell death	4.23E-25	620	cell death	8.61E-19	385	cell death	2.17E-23	215	cell death	1.93E-09	222
	apoptosis of tumor cell lines	2.58E-20	259	cell death of tumor cell lines	2.31E-20	213	cell death of tumor cell lines	1.47E-32	139			
	advanced malignant tumor	7.90E-22	184	apoptosis of tumor cell lines	3.50E-20	178	apoptosis of tumor cell lines	5.50E-28	117			
	invasion of cells	4.59E-20	193									
	cell movement	2.37E-19	396									
	proliferation of tumor cell lines	2.34E-18	299									
3	cell death of tumor cell lines	1.01E-24	328	breast or colorectal cancer	1.33E-21	484	epithelial neoplasia	1.11E-29	319	benign neoplasia	1.84E-08	67
	necrosis of epithelial tissue	8.07E-19	149	breast or ovarian cancer	1.51E-19	220	carcinoma	1.76E-28	312	epithelial neoplasia	2.90E-08	201
	cell survival	9.11E-19	286	abdominal neoplasm	8.96E-19	655	solid tumor	5.52E-28	313	solid tumor	3.64E-08	196
	cell viability of tumor cell lines	3.29E-18	173	epithelial neoplasia	1.34E-18	672	Cancer	4.66E-27	346	carcinoma	3.76E-08	192
	abdominal neoplasm	6.37E-25	1070	carcinoma	3.05E-18	653	metastasis	5.95E-24	73	growth of tumor	1.40E-07	41
	Cancer	1.20E-23	1499	abdominal cancer	9.78E-18	640	growth of tumor	1.84E-20	55	Cancer	1.52E-07	258
	epithelial neoplasia	3.43E-19	1072	Cancer	1.20E-17	907						
	digestive tract cancer	8.56E-21	771	metastasis	1.23E-14	113						
				precancerous condition	3.09E-14	395						
	abdominal cancer	9.74E-23	1042									
4	breast or colorectal cancer	1.33E-22	755	Gastrointestinal Tract Cancer and Tumors	7.79E-16	431	digestive organ tumor	1.88E-26	147			
	metastasis	4.88E-22	183	gastrointestinal tumor	1.67E-15	431	intestinal cancer	1.30E-19	100			
				digestive organ tumor	2.71E-15	478	gastrointestinal tract cancer	2.58E-19	106			
				gastrointestinal tract cancer	4.15E-15	425	colorectal tumor	1.17E-19	100			
				digestive tract cancer	1.79E-14	469						
				intestinal tumor	1.96E-14	395	cell movement	8.82E-32	169	invasion of cells	1.25E-14	81
5	carcinoma	1.77E-19	1044	cell movement	3.48E-14	246	migration of cells	2.05E-29	154	invasion of tumor cell lines	4.97E-13	62
	organismal death	8.37E-21	455				cell movement of tumor cell lines	6.97E-25	84	migration of cells	2.13E-10	139
	invasion of tumor cell lines	1.20E-18	148				invasion of cells	7.56E-24	83	invasion of melanoma cell lines	1.05E-09	17
							invasion of tumor cell lines	3.26E-21	65	cell movement	1.49E-09	148
							migration of tumor cell lines	3.36E-21	68	invasion of breast cancer cell lines	2.29E-08	27
6	digestive organ tumor	1.19E-20	779	mammary tumor	2.14E-14	189						
	mammary tumor	4.52E-19	300				proliferation of tumor cell lines	2.95E-20	117	proliferation of tumor cell lines	4.92E-09	113
				cell cycle progression	7.46E-15	145						
				gonadal tumor	7.47E-15	97	development of blood vessel	1.67E-20	82	cell movement of endothelial cells	1.36E-09	40
							vasculogenesis	1.25E-19	75	vasculogenesis	4.01E-09	67
										development of blood vessel	9.10E-09	72
										migration of endothelial cells	1.16E-08	36
										adhesion of connective tissue cells	1.70E-07	21
										cell spreading	3.18E-08	33
										organization of cytoskeleton	1.32E-07	95
										chronic kidney disease	1.48E-07	18

1 Cellular Growth and Proliferation

2 Cell Death and Survival

3 Cancer

4 Cancer, Gastrointestinal Disease

5 Cellular Movement

6 Others including Cancer, Organismal Injury and Abnormalities, Reproductive System Disease, Cellular Development, Cellular Growth and Proliferation, and Organismal Survival for MDA-MB-231, or Cancer, Organismal Injury and Abnormalities, Reproductive System Disease, Cell Cycle, and Cancer, Endocrine System Disorders, Organismal Injury and Abnormalities, Reproductive System Disease for MCF-7

After IPA, the Canonical Pathways predicted with statistical significance after HERV-K knockdown are shown in Figure S4A for the BC cell lines (MDA-MB-231, MCF-7, and SKBR3) and in Figure S4B for the tumor biopsies (MDA-MB-435.eB1 and SKBR3). Signaling networks of MDA-MB-231 and MCF-7 cell lines show a major downregulation of EGFR and NF-kB (Figure S4C). Overexpression of EGFR has been demonstrated in approximately half of cases of triple-negative breast cancer and inflammatory breast cancer. EGFR and its downstream pathway regulate epithelial–mesenchymal transition (EMT), migration, and tumor invasion [13]. NF-kB plays a key role in regulating the immune response to infection and promotes breast cancer tumor-initiating cells [14]. Signaling networks of SKBR3 and MDA-MB-435.eB1 cell lines (Figure S4D) show a major convergence on ERK1/2 after HERV-K knockdown, while the network from SKBR3 (tumor biopsies or cell lysis; Figure S4E), showed a centering on MYC. Both the ERK1/2 and MYC networks play key roles in cancer development.

IPA revealed that, for the 4 BC cell lines evaluated, the transcriptional regulators p53 and MYC were the major upstream regulators influenced by HERV-K knockdown (Table 1). Transforming growth factor, β1 (TGFB1) was by far the most common growth factor upstream regulator in all 4 BC cell lines that was impacted by HERV-K knockdown. β-estradiol was the major endogenous chemical, and dexamethasone the major drug, affected by HERV-K knockdown. The major cytokine controlling changes in gene expression after HERV-K knockdown was TNF, the major kinase was ERBB2, and the major oncogene was HRAS and/or KRAS. Other significant upstream pathway changes associated with HERV-K *env* knockdown were ERK, ESR1, and MEK.

IPA disease and functional endpoints identified several categories of disease and cell function affected by shRNA knockdown of HERV-K. In the Cellular Growth and Proliferation category, proliferation of cells was the main endpoint affected. Endpoints affected by knockdown in the Cell Death and Survival category included cell death, apoptosis, and necrosis. In the Cancer category, cancer, epithelial neoplasia, and metastasis were the endpoints influenced by HERV-K knockdown, and in the Cellular Movement category, cell movement and invasion of tumor cell lines were the major endpoints affected by HERV-K knockdown (Table 2).

### Phosphorylation profiles of kinases in HERV-K knockdown cells

Cellular extracts prepared from the MCF-7 cell line transduced with shRNAenv vs. shRNAc were compared using phosphoprotein arrays.The top ten upregulated proteins in MCF-7 cells transduced with shRNAenv compared with shRNAc were p38a, MSK1/2, Fgr, HSP27, TOR, EGFR, AMPKα1, Akt1/2/3 S473, STAT3 Y705 and STAT2, and the top ten downregulated proteins were eNOS, RSK1/2/3, HSP60, SAR5a/b, Fyn, Lck, Lyn, p70 S6 Kinase, Hck, and Akt1/2/3 T308. The phosphorylation profiles of kinases and their protein substrates in MCF-7 cells transduced with shRNAenv vs. shRNAc are shown in Table 3.

**Table 3:** The phosphorylation profiles of kinases and their protein substrates in MCF-7 cells transduced with shRNAenv vs. shRNAc

MCF-7	shRNAc	shRNAenv	Ratio
eNOS	1	0.11064	0.11064182
RSK1/2/3	1	0.62695	0.6269543
HSP60	1	0.67656	0.67656339
STAT5a/b	1	0.67902	0.67901655
Fyn	1	0.74091	0.74091294
Lck	1	0.77592	0.77591621
Lyn	1	0.78501	0.78500609
p70 S6 Kinase	1	0.7877	0.78769829
Hck	1	0.80348	0.80347775
Akt 1/2/3 T308	1	0.81457	0.81457277
STAT3 S727	1	0.81635	0.8163504
β-Catenin	1	0.82757	0.82756674
FAK	1	0.83127	0.83127401
PDGF Rβ	1	0.86031	0.86030859
Chk-2	1	0.88314	0.88314166
CREB	1	0.92764	0.92763798
STAT5a	1	0.94686	0.94686027
Yes	1	0.96352	0.96351656
STAT5b	1	0.97903	0.97902745
PRAS40	1	0.97989	0.97989347
p53 S392	1	1.00648	1.00648388
p53 S46	1	1.01919	1.01918852
GSK-3α/β	1	1.02083	1.02082839
c-Jun	1	1.02129	1.02129322
JNK 1/2/3	1	1.02866	1.02865523
p53 S15	1	1.04628	1.04627875
ERK1/2	1	1.04683	1.04683285
WNK1	1	1.05737	1.0573731
AMPKα2	1	1.0578	1.05779985
Src	1	1.06922	1.06921719
STAT6	1	1.07413	1.07412904
STAT2	1	1.09571	1.09571017
STAT3 Y705	1	1.10981	1.10981287
Akt 1/2/3 S473	1	1.15316	1.15316022
AMPKα1	1	1.15757	1.15756881
EGF R	1	1.23593	1.23592759
TOR	1	1.2745	1.27449869
HSP27	1	1.28963	1.2896269
Fgr	1	1.31598	1.31597666
MSK1/2	1	1.41262	1.41262168
p38α	1	1.70822	1.70821786

### Influence of HERV-K knockdown on expression of Ras and EMT markers in BC cell lines

**Figure 5:**
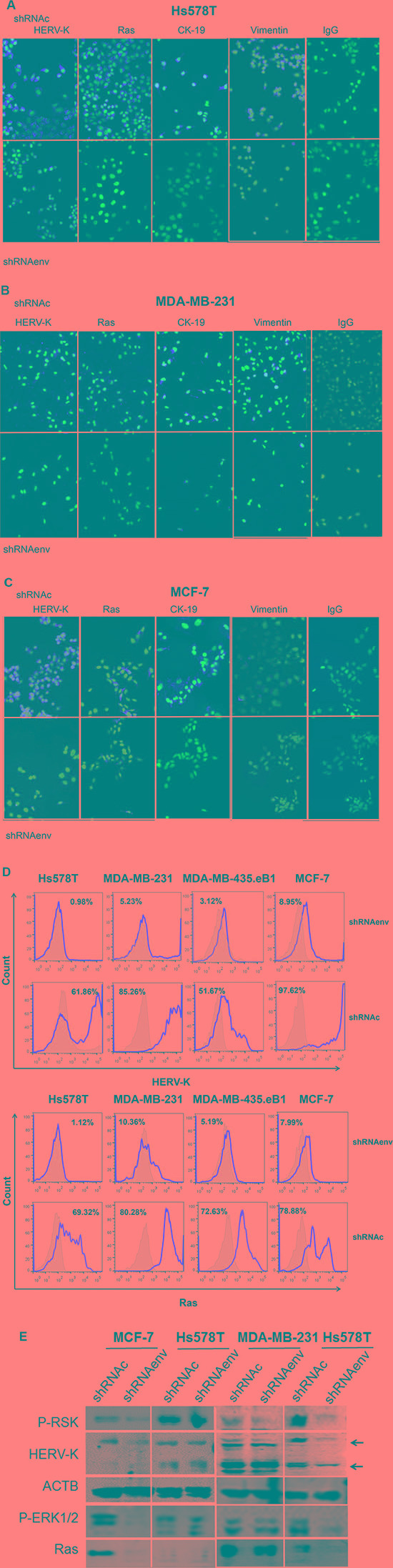
Changes in expression of signaling proteins after shRNA knockdown of HERV-K IFS was used to detect changes in expression of HERV-K and Ras in Hs578T (**A**) MDA-MB-231 (**B**), and MCF-7 cells (**C**) after HERV-K knockdown. The effect of HERV-K knockdown on expression of proteins involved in EMT, including CK-19 and vimentin was determined in the three BC cell lines. Reduced protein expression was observed for all of these proteins. In addition, decreased Ras protein expression (D, left) was induced by downregulation of HERV-K expression (D, right) in cancer cells transduced with shRNAenv, as assessed by FACS (**D**). Immunoblot analysis showed reduced expression of p-RSK, p-ERK 1 or 2, and Ras in shRNAenv transduced MCF-7, MDA-MB-231, and Hs578T cells only when there was also shRNAenv-induced reduced expression of HERV-K Env protein in these cell lines (**E**). ACTB antibody was used as control.

IFS was employed to detect the expression of HERV-K Env protein, Ras (antibody 27H5, which detects endogenous levels of total K-, H-, and N-Ras proteins), and EMT markers in cells after HERV-K knockdown by shRNAenv. Knockdown of HERV-K Env expression was accompanied by downregulation of Ras protein expression in three BC cell lines (Figure 5A–5C). To evaluate if this change was the result of an EMT, we tested for expression of EMT markers. HERV-K shRNA transduction led to decreased expression of CK19 and vimentin in the three BC cell lines. These data indicate that the phenotype change associated with shRNAenv treatment was not the result of an EMT, because shRNAenv led to reduced expression of both the characteristic mesenchymal marker vimentin and the characteristic epithelial marker CK-19.

### Downregulated expression of HERV-K *env* RNA blocked signaling via the Ras/Raf/MEK/ERK pathway

Strong and significant reduction of K-Ras expression in SKBR3 (8.38E-10), MDA-MB-231 (3.14E-12), MDA-MB-435.eb1 (1.26E-06) and MCF-7 (4.19E-19) cells, and of H-Ras in MDA-MB-435.eb1 (1.19E-10), MCF-7 (3.14E-11), SKBR3 (7.48E-15), and MDA-MB-231 (1.15E-18) cells after HERV-K knockdown was revealed by RNA-Seq assays (Table 1). Our RNA-Seq and IFS staining results suggested some important potential targets of HERV-K in BC cells, prompting us to pursue an investigation of changes in Ras/Raf/MEK/ERK pathway signaling resulting from knockdown of HERV-K.

A greater degree of HERV-K Env protein downregulation was associated with a correspondingly greater decrease in the expression of Ras in Hs578T, MDA-MB-231, MDA-MB-435.eB1, and MCF-7 cells (Figure 5D), as assessed by FACS. Reduced expression of HERV-K after knockdown with shRNAenv was associated with decreased expression of p-RSK, p-ERK1/2, and Ras in the three BC cell lines (Figure 5E and S5A), as assessed by immunoblot. HIF-1α is also a driver of EMT, and its expression was downregulated in the 3 shRNAenv-transduced BC cell lines compared with shRNAc by immunoblot (Figure S5A). HIF-1α expression is associated with increased proliferation, and has an adverse prognostic impact with increased concentrations in invasive ER-positive breast cancer [15]. Downregulation of HIF-1α expression by shRNA inhibited proliferation, migration, and invasion of cancer cells *in vitro*, and decreased tumor growth in breast cancer xenograft models [16]. In addition, there was increased expression of EpCAM in MCF-7 and Hs578T cells transduced with shRNAenv (data not shown), but during the progression of EMT both EpCAM and CK are downregulated.

### Upregulated expression of HERV-K induced BC cell invasion and migration

**Figure 6:**
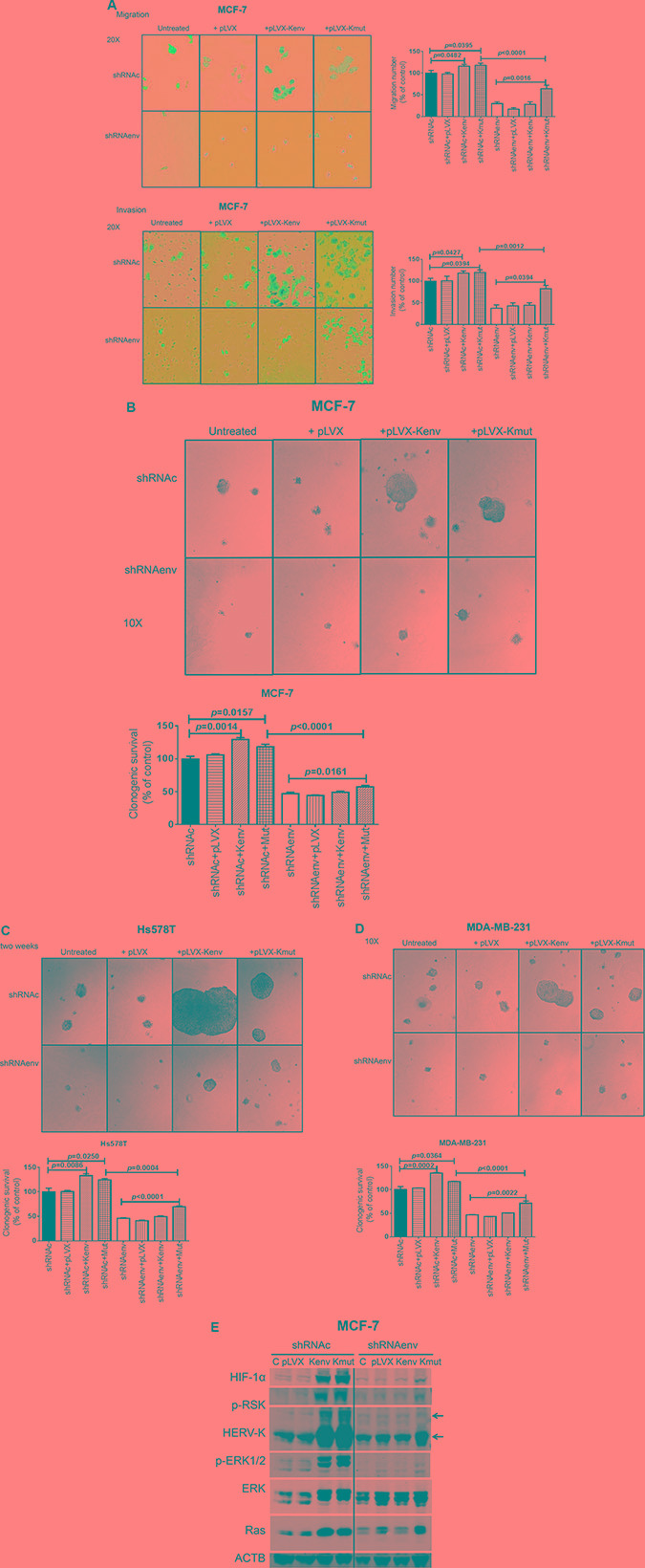
Effect of overexpression of HERV-K on *in vitro* migration, invasion, and transformation in cancer cells Significantly decreased migration (*p* < 0.0001; (A, top right) and invasion (*p* < 0.0001; **A**, bottom right) of MCF-7 cells stably transduced with shRNAenv was observed, compared with cells transduced with a scrambled shRNAc vector. Migration and invasion was increased if shRNAc cells were transduced with an HERV-K env expression vector [+Kenv; *p* = 0.0482 for migration (A, top right) and *p* = 0.0427 for invasion (A, bottom right)] or a vector expressing a mutated env gene [+Kmut; *p* = 0.0395 for migration (A, top right) and *p* = 0.0394 for invasion (A, bottom right) (*n* = 11; unpaired *t* test)]. Untreated and vector only (+pLVX) were used as controls. No significant change was observed in shRNAenv cells transduced with Kenv, likely due to continued shRNAenv knockdown in these cells even in the presence of higher levels of Kenv. Significant change was observed in shRNAenv cells transduced with Kmut for migration (*p* = 0.0016) and invasion (*p* = 0.0394). Anchorage-independent growth assays were employed to determine cell transformation after BC cells were transduced with HERV-K env or mutated env gene. Images were taken 2 weeks post-seeding of cells in soft agar. Larger colonies and significantly increased numbers of colonies were observed for shRNAc cells after transduction with Kenv or Kmut in MCF-7 shRNAc (*p* = 0.001 and *p* = 0.016, respectively; Figure B), Hs578T shRNAc (*p* = 0.0086 and *p* = 0.025, respectively; Figure C), and MDA-MB-231 (*p* = 0.0002 and *p* = 0.036, respectively; Figure D) cells. Significantly increased numbers of colonies were observed for shRNAenv cells transduced with Kmut in MCF-7 (**B**: *p* = 0.001), Hs578T (**C**; *p* < 0.0001), and MDA-MB-231 (**D**; *p* = 0.002) cells (*n* = 4; unpaired *t* test). (**E**) Immunoblot assays showed increased levels of HIF-1alpha, p-RSK, HERV-K, p-ERK 1 or 2, and Ras protein in MCF-7 shRNAc cells transduced with Kenv or Kmut and in MCF-7 shRNAenv cells transduced with Kmut. The deviation (error bars) represents standard error of the mean (SEM).

Since downregulated expression of HERV-K *env* RNA reduced the migration and invasion of BC cells, we further investigated the changes in these processes after restoring the expression of HERV-K *env* by transducing cells with a HERV-K *env* expression vector (+pLVX-Kenv; Figure S5B) or a vector with a synonymous mutant of HERV-K *env* (+pLVX-Kmut), whose purpose was to show specificity for shRNAenv, since the shRNAenv cannot knock down Kmut. Vector only (+pLVX) or untreated cells were used as controls. Increased migration was demonstrated in MCF-7 shRNAc cells transduced with either pLVX-Kenv (*p* = 0.048) or pLVX-Kmut (*p* = 0.039), compared to untreated cells or cells with added vector only (+pLVX) (Figure 6A, top left and top right panels). However, migration was increased only in MCF-7 shRNAenv cells with added pLVX-Kmut (*p* = 0.002), and not in MCF-7 shRNAenv cells transduced with the non-mutated expression vector pLVX-Kenv. Similarly, increased invasion was demonstrated in MCF-7 shRNAc cells with added pLVX-Kenv (*p* = 0.043) and pLVX-Kmut (*p* = 0.039), compared with untreated cells or cells with added vector only (+pLVX), but invasion was increased only in MCF-7 shRNAenv cells with added pLVX-Kmut (*p* = 0.001), and not in MCF-7 shRNAenv cells with added pLVX-Kenv (Figure 6A, bottom left and bottom right panels).

### Increased transformation of BC cells after HERV-K *env* transduction

We further investigated BC cell transformation using an anchorage-independent growth assay, after shRNAenv or shRNAc cells had been transduced with HERV-K *env* gene. Larger colonies were observed in shRNAenv or shRNAc MCF-7 (Figure 6B, top panel), Hs578T (Figure 6C, top panel), and MDA-MB-231 (Figure 6D, top panel) cells transduced with pLVX-Kenv and pLVX-Kmut, in comparison to cells transduced with control plasmids that did not express HERV-K. When comparing shRNAc cells with shRNAenv cells, shRNAc cells formed larger colonies than shRNAenv transduced with pLVX-Kenv and pLVX-Kmut (Figure 6B–6D, top panel). Significantly reduced colony numbers were observed in the cells transduced with shRNAenv compared with shRNAc (*p* = 0.0004 for Hs578T, *p* < 0.0001 for MCF-7, and for MDA-MB-231 cells). In contrast, significantly increased colony numbers were observed in shRNAenv or shRNAc cells transduced with pLVX-Kmut and in shRNAc cells transduced with pLVX-Kenv. MCF-7 cells (derived from adenocarcinoma metastatic site) were not as invasive as MDA-MB-231 cells in our assay (Figure 6A), and MCF-7 cells showed no metastasis in animal xenograft models (data not shown). However, when we overexpressed HERV-K the invasion in this cell line increased substantially, which may in part explain why HERV-K promotes invasion and metastasis. Of great importance, knockdown of HERV-K with shRNAenv reversed this increase in invasiveness that was induced by overexpression of HERV-K (+pLVX-Kenv), but not by overexpression of HERV-K with mutation (+pLVX-Kmut).

Immunoblotting revealed increased expression of HERV-K Env protein and other proteins including HIF-1α, p-RSK, p-ERK1/2, and Ras in shRNAc cells transduced with pLVX-Kenv and pLVX-Kmut (MCF-7 cells, Figure 6E and Figure S5C). However, increased expression of these proteins was only observed in the shRNAenv cells with added pLVX-Kmut, and not in shRNAenv cells transduced with pLVX-Kenv.

### Increased metastasis of BC cells expressing the HERV-K *env* gene *in vivo*

**Figure 7:**
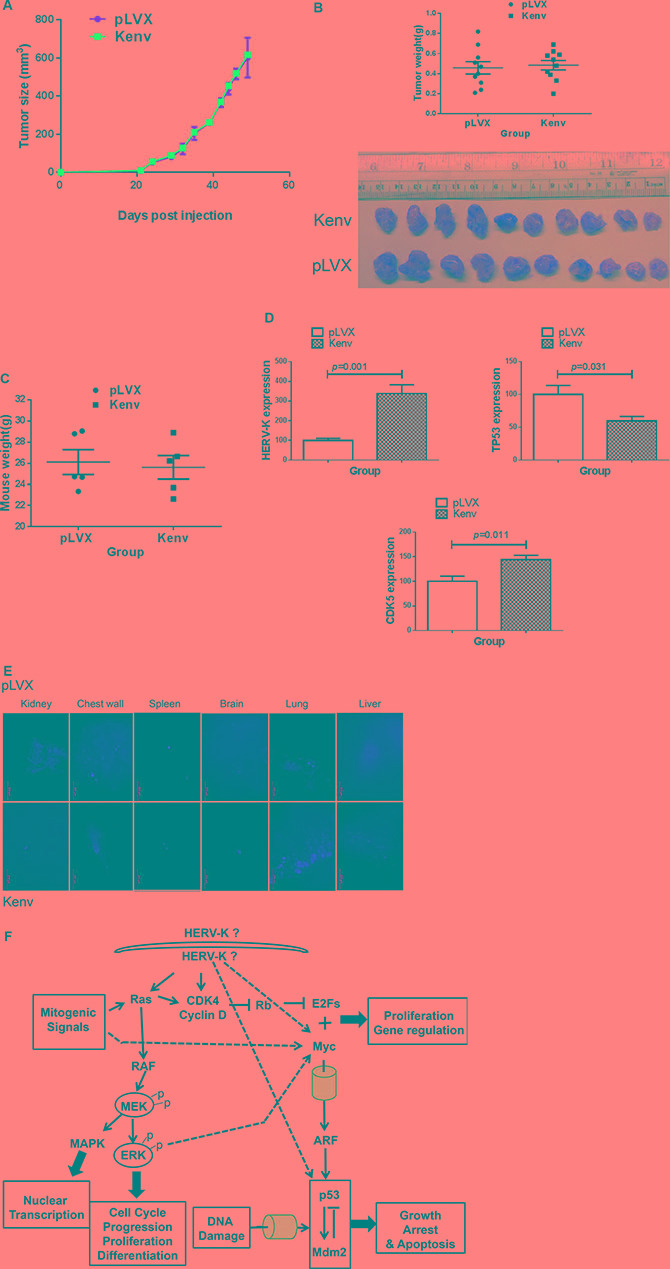
Effect of HERV-K overexpression on tumor growth in mouse xenografts No significant increase in tumor growth was observed in immunodeficient mice xenografted with MDA-MB-231 cells transduced with Kenv compared with cells transduced with pLVX (vector only) (**A**). Tumor sizes and weights were not significantly increased in xenografts of cells transduced with Kenv (*p* = 0.7397) (**B**). A slight but nonsignificant loss in body weight was observed in mice bearing Kenv cells compared with pLVX cells (*p* = 0.7635) (**C**), and qRT-PCR was employed to determine the expression of HERV-K in MDA-MB-231+Kenv or +pLVX. Significantly increased expression of HERV-K and CDK5 in MDA-MB-231+Kenv cells was observed, compared with MDA-MB-231+pLVX cells (**D**). There was also significantly reduced expression of p53 in MDA-MB-231+Kenv cells (D). Increased metastasis to lung (red fluorescence) in MDA-MB- 231+Kenv cells was observed (**E**). A summary of pathways related to HERV-K Env protein is shown (**F**).

No significant change in tumor growth and weights was seen in xenografts of MDA-MB-231 cells stably transfected with pLVX-Kenv vs. vector only (pLVX) (Figure 7A and 7B). Body weight was similarly unaffected in these mice bearing tumors expressing HERV-K (Figure 7C); there was a trend toward increased brain, spleen, and liver weight in these mice (Figure S6A–S6C). However, no significant differences between the groups in other organs were detected (Figure S6D and S6E). Of interest, upregulated expression of HERV-K accompanied downregulated expression of p53 and upregulated expression of CDK5 in these MDA-MB-231 +Kenv tumors, compared with the same cells + pLVX controls (Figure 7D). More metastasis to lung was detected in lung after HERV-K overexpression (Figure 7E). A summary of pathways related to HERV-K Env protein is shown in Figure 7F.

## DISCUSSION

The current study has provided strong evidence for an important role of HERV-K in human tumorigenesis. This is in agreement with several recent studies documenting key functions of HERVs in sensitization to immunotherapy [17], targeting of cancer-initiating cells [18], and promoting cytolytic activity in multiple tumor types [19].

Our RNA-Seq data support the role(s) of HERV-K *env* expression in promoting BC tumorigenesis by providing links with p53, Myc, and Ras signaling pathways. Our pathway analysis of HERV-K knockdown BC cell lines, based on the RNA-seq data, demonstrates that HERV-K facilitates maintenance of the transformed phenotype and promotes metastasis by promoting key signaling pathways involved in cellular movement, cancer, cell death/survival, and cell growth/proliferation. The data obtained from the phosphorylation profiles of kinases in HERV-K knockdown BC cells further support the involvement of the signaling pathways identified by our RNA-Seq analyses.

In a previous study we reported that treatment of BC cells with an anti-HERV mAb impacted p53 signaling pathways [6], a finding supported by our recent HERV-K-chimeric antigen receptor (K-CAR) studies showing that HERV-K affects tumorigenesis via a similar mechanistic effect on p53 signaling [8]. Our RNA-seq results additionally suggest important roles of TGF-β1, β-estradiol, dexamethasone, TNF, and ERBB2 in mediating the effect of HERV-K knockdown in BC cells. There is little in the literature linking TGF-β1to endogenous retroviruses, although our RNA-Seq data suggest that it plays a very important mechanistic role in mediating HERV signaling in BC, and that the relationship between HERV-K and TGF-β1in BC is deserving of further study. It has already been established by us and others that female hormones including β-estradiol increase the expression of HERV-K [3, 20]. We previously reported that there was a greatly increased frequency of TNF-α–secreting CD8+ T cells in HERV-K–stimulated PBMCs from BC patients, indicating a role for HERV-K in TNF-α secretion [5]. HERV-K and ERBB2 are both expressed in BC, but there has not been a detailed analysis of their co-expression in BC, and we have found a much broader expression pattern of HERV-K than that of ERBB2 in BC.

The Ras/Raf/MEK/ERK pathway is known to play a pivotal role in differentiation, proliferation and tumor progression. HERV-K Env protein downregulation correlated with downregulation of p-ERK protein in most of the mice treated with K-CAR T cells [8]. In an earlier study, knockdown of wild-type p53 led to activation of p-ERK1/2 [21], showing a reciprocal relationship similar to what we observed in the current study. The Ras/Raf/MEK/ERK signaling pathway has been proposed to play an important role in HERV-K activation [22]. These observations may thus provide a mechanistic explanation of the role(s) HERV-K plays in breast tumorigenesis.

The link between HERV-K and Ras signaling was more firmly established in this study. Ras genes are among the most frequently mutated proto-oncogenes in cancer, and Ras proteins (K-Ras, H-Ras, and N-Ras) are known to regulate a number of processes that govern cancer progression, including cell proliferation, transformation, differentiation, and survival [23, 24]. However, how Ras stability is regulated remains largely unknown. Ras mutants trigger activation of downstream signaling pathways, thereby promoting cancer development. The MAPK/ERK pathway and PI3K/Akt pathway are two essential components in Ras-induced transformation and tumorigenesis [25]. Although Ras mutations are very rare in human BCs, the Ras signaling pathway is hyperactivated in half of these tumors [26], and most of the effector pathways activated by Ras mutations promote cell growth and contribute to malignant transformation [27, 28]. Ras may be more frequently activated by other mechanisms in these tumors, but the mechanism(s) by which Ras becomes activated in BC has remained elusive [26]. Although Ras mutations are rare in BC, amplifications of wild type Ras are frequently observed in basal BCs [29], the most aggressive subtype of human BC, underscoring the connection between Ras activation and BC progression.

Previously, we and others have reported that HERV-K Env protein may serve as an oncogene that promotes tumor proliferation and metastasis in BC and other cancers. Our current study provides convincing evidence that HERV-K specifically increases Ras-induced ERK activation, and suggests that the oncogenic activity of Ras protein is propagated by activation of HERV-K Env protein. Importantly, the expression of HERV-K Env protein in metastatic tumor tissues treated with K-CAR T cells correlated with the expression of Ras, and this HERV-K-specific CAR prevented tumor metastasis to other organs. Furthermore, downregulation of HERV-K expression in tumors of mice treated with K-CAR correlated with upregulation of p53 and downregulation of MDM2, p-ERK, and Ras [8].

Because overexpression of HERV-K is essential for promoting tumorigenesis, and shRNA knockdown of HERV-K expression eliminates the tumorigenic effect of HERV-K in Ras-initiated cancers, our studies reveal a fundamental role of HERV-K Env protein in regulating the Ras signaling pathway. Activation of HERV-K Env protein thus appears to be a critical first step in Ras-induced transformation and tumorigenesis of human BC cells.

The decrease in Ras expression that resulted from HERV-K knockdown with an shRNA was associated with phenotypic changes that resembled the development of EMT. But proteins whose expression changed in HERV-K knockdown cells were not characteristic of EMT. We hypothesize that HERV-K knockdown BC cells may be undergoing a reversion to a non-tumorigenic phenotype, a hypothesis supported by both *in vitro* and *in vivo* data.

In this study we investigated aspects of HERV-K expression that promoted BC progression. Significantly decreased migration, invasion, and transformation was documented in BC cell lines stably transduced with a vector that knocked down expression of the HERV-K *env* gene. Knockdown of HERV-K in these cells by introduction of an shRNA that blocked its expression also led to decreases in tumor growth and tumor weight, as well as downregulated expression of pERK1/2 and Ras proteins.

Our current study and a previous study using a chimeric antigen receptor antibody demonstrated that expression of HERV-K is related to breast cancer metastasis [8]. Since immunodeficient mice bearing MDA-MB-231 tumors consistently develop widespread metastases, we decide to evaluate this xenograft model initially, with a focus primarily on metastasis. Our results show that overexpression of HERV-K in this metastatic breast cancer cell line does indeed lead to increased lung metastasis. With respect to tumor growth, we suggest that the aggressive MDA-MB-231 tumors have reached their maximum potential for growth in xenografts, and overexpression of HERV-K does not further promote tumor growth in this model. However, HERV-K overexpression does still stimulate metastasis in this model. In future and ongoing studies we are focusing our xenograft studies on cells that have lower expression of HERV-K, such as conditionally reprogrammed normal breast, immortalized breast, and pre-neoplastic breast cells, to determine whether HERV-K overexpression from a baseline of low expression will promote tumorigenesis in mouse xenograft models of breast cancer.

Although mice inoculated with MDA-MB-231 cells stably transduced with the HERV-K *env* gene (Kenv) did not show increased tumor weights, there was increased *in vitro* invasion, migration, and colony formation in soft agar, all of which support a role of HERV-K in tumor progression and metastasis. This concept was supported by our *in vivo* observation of increased metastasis to the lung and brain in mice whose tumors overexpressed HERV-K. Overexpression of CDK5 in breast cancer has been found to correlate with several poor prognostic parameters. It is essential for TGF-β-induced EMT and breast cancer progression [30].

In conclusion, our data indicate that the expression of HERV-K Env protein is essential for Ras-induced tumorigenesis in BC cells. We also provide evidence that downregulation of HERV-K expression by shRNA can be used for understanding mechanisms of tumorigenesis of BC, and that HERV-K knockdown may induce reversion of BC cells to a non-tumorigenic phenotype. HERV-K is thus a novel target for therapeutic intervention of Ras-driven tumors. Our studies have identified an important tumor oncogene involved in BC progression and have revealed an alternative mechanism by which Ras becomes activated in this disease.

## MATERIALS AND METHODS

### Cell culture

All BC cell lines (MDA-MB-231, Hs578T, MCF-7, SKBR3 and 293TN cells) were purchased from American Type Culture Collection (ATCC; Manassas, VA, USA). MDA-MB-231 (metastatic; 51 year old Caucasian female) and Hs578T (74 year old Caucasian female) are triple negative BC cell lines. MCF-7 (estrogen receptor positive; 69 year old Caucasian female) and SKBR3 (overexpressing HER2; 43 year old Caucasian female) are BC cell lines derived from an adenocarcinoma metastatic site. MDA-MB-435.eB1 cells (31 year old Caucasian female: a gift from Dr. Michael Rosenblum) are human BC cells that have been transfected with c-erbB2 oncogene, and express high levels of the trans-membrane glycoprotein p185 c-erbB2. Cells were cultured in RPMI or DMEM (Thermo Scientific, Rockford, IL) with 10% FBS (Thermo Scientific) and 5% glutamax (Gibco Life technologies, Grand Island, NY) (complete media). All cell lines were mycoplasma free in our experiments. Cell viability was analyzed before each assay using a trypan blue dye exclusion assay, and was always greater than 96%.

### HERV-K *env* shRNA lentiviral packaging and transduction

Short hairpin RNAs (shRNAs) targeting the HERV**-**K *env* gene (GenBank No. M14123.1) (shRNAenv) and matched scrambled shRNA sequences serving as negative controls (shRNAc) were designed using the Invitrogen RNAi Designer program, and cloned into the pGreenPuro™ lentiviral expression vector (System Biosciences)(SBI). The shRNA**-**expressing lentiviral particles were then packaged using pPACKH1 Lentivector Packaging Kit (SBI) following the manufacturer's instructions, and the viral particles were generated in our laboratory. Virus particles were titered using H1299 cells following the manufacturer's instructions. BC cells (1 × 10^5^) were seeded into individual wells of 6**-**well plates and infected with lentiviral particles carrying shRNAenv or matched shRNAc at a multiplicity of infection (MOI) of 40.

### RT-PCR and qRT-PCR analysis

RNA was isolated from cells or tumor biopsy specimens [2, 31]. qRT-PCR was carried out using the TaqMan^®^ One-Step RT-PCR Master Mix Reagents Kit (Life Technologies, Grand Island, NY) to amplify a HERV-K specific sequence, as described previously [3]. Multiple HERV-K gene fragments were amplified by RT-PCR using the QIAGEN OneStep RT-PCR Kit (QIAGEN, Valencia, CA): RT-PCR was carried out by reverse transcription for 30 min at 50ºC, followed by 15 minutes of inactivation at 95ºC, and 35 cycles of 30 seconds at 94ºC, 30 seconds at 55ºC, and 60 seconds at 72ºC for both types of HERV-K surface (SU) genes.

### Immunoblot analysis

Total protein lysates of cells or tissues were used for immunoblot analysis (25 μg of protein per lane) as described previously [5, 32]. Anti-HERV-K mAb 6H5 (1 μg/ml) generated from our lab [5, 6], anti-actin (ACTB) antibodies (1:100 dilution; University of Iowa), phospho-p44./p\42 MAP kinase (Erk1/2) (137F5) (Cell Signaling Technology), HIF-1α (1:1,000 dilution; Santa Cruz), anti-Ras antibody (1:1,000 dilution; 27H5; Rabbit mAb #3339; Cell Signaling Technology), and MDM2 (1:1,000 dilution; Sigma-Aldrich) were used as primary antibodies. Anti-mIgG-680 or anti-rIgG-680 fluorescent antibody (1:5000 dilution; Li-Cor, Lincoln, NE) served as the secondary antibody, as described previously [33, 34], and signal was detected and recorded by the Li-Cor Odyssey imaging system. Protein levels were quantified by Image J software (NIH, Bethesda, MD).

### Alamar blue assay for cell viability and cell proliferation

BC cells were seeded in flat-bottomed 96-well culture plates at a density of 1 × 10^4^ cells/well. After 24 hours, ten microliters of Alamar Blue (AbD Serotec, U.K.) was added to each well and the plates were incubated 37°C and fluorescence intensity was measured at different time points using a Victor 1402 multilabel counter (Wallac). The amount of fluorescence was a measure of cell viability.

For cell proliferation assays by cell counting, 1 × 10^5^ cells per well were plated in complete medium in a 24**-**well plate and incubated at 37°C. Cells were then harvested, at different time points and proliferation rates were measured by counting viable cells using the trypan blue dye exclusion method.

### Anchorage-independent growth

Anchorage-independent growth of shRNA transduced cells was tested according to a published method [35]. Colonies were counted after 2 to 3 weeks of incubation and scored by counting the number of colonies greater than 50 μm in diameter.

### Transwell plate assay

For migration and invasion assays, 24-well plates with transwell permeable supports (Fisher Scientific, Pittsburgh, PA) were used. Cells were trypsinized, counted and suspended in serum-free medium at a dilution of 2.5 × 10^5^/ml. 200 μl serum-free medium with suspended cells was placed into the upper chamber, which was coated with Matrigel (BD Biosciences, San Jose, CA) for invasion assays or left uncoated for migration assays. 750 μl complete medium was placed into the bottom chamber. This assay, as well as time-lapse wound-healing assays (scratch assay), were completed using a standard protocol [36].

### *In vivo* studies

Female immunodeficient nude or NOD/SCID/gamma mice, 6- to 8-weeks of age (NCI, Frederick, MD), were inoculated subcutaneously in the flank with 1 × 10^6^ MDA-MB-231, MDA-MB-435.3B1, or SKBR3 cells transduced with shRNAenv or shRNAc to assess tumorigenesis and phenotypes of these cells *in vivo*. Tumor growth was measured twice per week. Tumors were harvested and weighed, H&E staining was used to provide histologic evidence of malignancy, and IHC was performed to analyze gene expression. RNAs were isolated from both groups and the expression of HERV-K *env* RNA was determined by qRT-PCR/RT-PCR. Lung tissues were also collected and cultured, and metastatic BC cells in the lung were compared between the two groups.

### Immunofluorescence staining and fluorescence-activated cell sorting assay

Cells were incubated with 6H5 mAb and analyzed by fluorescence microscopy to detect immunofluorescent staining of HERV-K conjugated with Alexa Fluor®488. DAPI (Invitrogen) was used for nuclear staining. For the detection of cytoplasmic Env expression, cells were treated with 0.1% Triton X-100 (Sigma-Aldrich) as described previously [12]. HERV-K SU protein levels were quantitated using the quantitative indirect immunofluorescence (QIFI) assay, with beads from a QIFIKIT kit (Dako North America, Inc., Carpinteria, CA), by flow cytometry using 6H5-A647 (antibody conjugated to Alexa Fluor dye), as described previously [6]. Propium iodide (PI) staining was performed for cell cycle analysis.

### RNA-seq and bioinformatics data analysis

RNA was isolated from the mouse xenograft tumors of shRNAenv or shRNAc cells. The RNA-seq libraries were prepared with an Illumina TruSeq Stranded Total RNA kit and sequenced using a 2 × 76 bases paired end protocol on the Illumina HiSeq 2000 instrument. Each library was sequenced in 1/6 lane, generating about 20–35 million pairs of reads per sample. The reads were mapped to human genome (hg19) by TopHat (V2.0.6). The number of fragments in each known gene from the RefSeq database (downloaded from UCSC Genome Browser on March 09, 2012) was enumerated using htseq-count from HTSeq package (V0.5.3p9). The differential expression was statistically accessed by R/Bioconductor package edgeR (V3.0.8). Genes with FDR ≤ 0.05 were called significant. All statistical tests were two-sided, and differences between variables with a *P* value of < 0.05 were considered statistically significant. The Ingenuity Pathway Analysis (IPA) program was used for downstream analysis of differentially expressed genes. The top 25 upstream regulators, and disease and functions from each paired cell line were used for analysis of the profile of each BC cell line.

### Immunohistochemistry for HERV-K Env expression and phosphorylation profiles of kinases in HERV-K knockdown cells

IHC was performed on 5 μm formalin-fixed, paraffin-embedded tissue sections using standard protocols and a Vectastain ABC Kit (Vector Laboratories, Burlingame, CA), as described previously [5]. A Human Phospho-Kinase Array (ARY003B; R&D systems) was employed to simultaneously detect the relative site-specific phosphorylation of 43 kinases. Cellular extracts prepared from the MCF-7 cell line transduced with shRNAenv vs. shRNAc were compared and results were evaluated after 30 min exposure.

### Construction of vectors expressing HERV-K *env* or HERV-K *env* mutant gene

A full-length HERV-K *env* sequence from a viral particle isolated from a BC patient diagnosed with invasive ductal carcinoma was cloned into vector pLVX-DsRed-Monomer-C1 to construct the HERV-K *env*-expressing vector pLVX-Kenv. Mutations that could eliminate the recognition of HERV-K *env* by shRNAenv but not influence the protein sequence of HERV-K Env were introduced into pLVX-Kenv using a QuikChange^®^ Site-Directed Mutagenesis Kit (Stratagene), to form pLVX-Kmut which could overcome the knockdown effect of shRNAenv if co-transduced with it. The HERV-K *env* expressing lentiviral particles were then packaged and titered in 293FT cells according to the manufacturer's instructions. The empty vector pLVX-DsRed-Monomer-C1 was also packaged to form the control lentiviral particles (pLVX). 1 × 10^5^ cells were seeded into individual wells of 6-well plates and infected with different lentiviral particles at a MOI of 40. After two weeks, qRT-PCR and immunoblot were performed to detect HERV-K Env expression.

### Statistical analysis

We used Student's *t***-**test to analyze differences between treatment groups in cell culture experiments using GraphPad Prism 6 (GraphPad Software). All statistical tests were two**-**sided, and differences between variables with a *P* value of < 0.05 were considered statistically significant.

## SUPPLEMENTARY MATERIALS FIGURES AND TABLES


