# Supplementary material for: Activation of HERV-K Env protein is essential for tumorigenesis and metastasis of breast cancer cells
Source: Oncotarget. 2016 Aug 20;7(51):84093–117. doi: 10.18632/oncotarget.11455 (PMC5356647; doi:10.18632/oncotarget.11455)
Supplement: Supplementary file 1 [file oncotarget-07-84093-s001.pdf]

Activation of HERV-K Env protein is essential for tumorigenesis and metastasis of breast cancer cells

Supplementary Materials

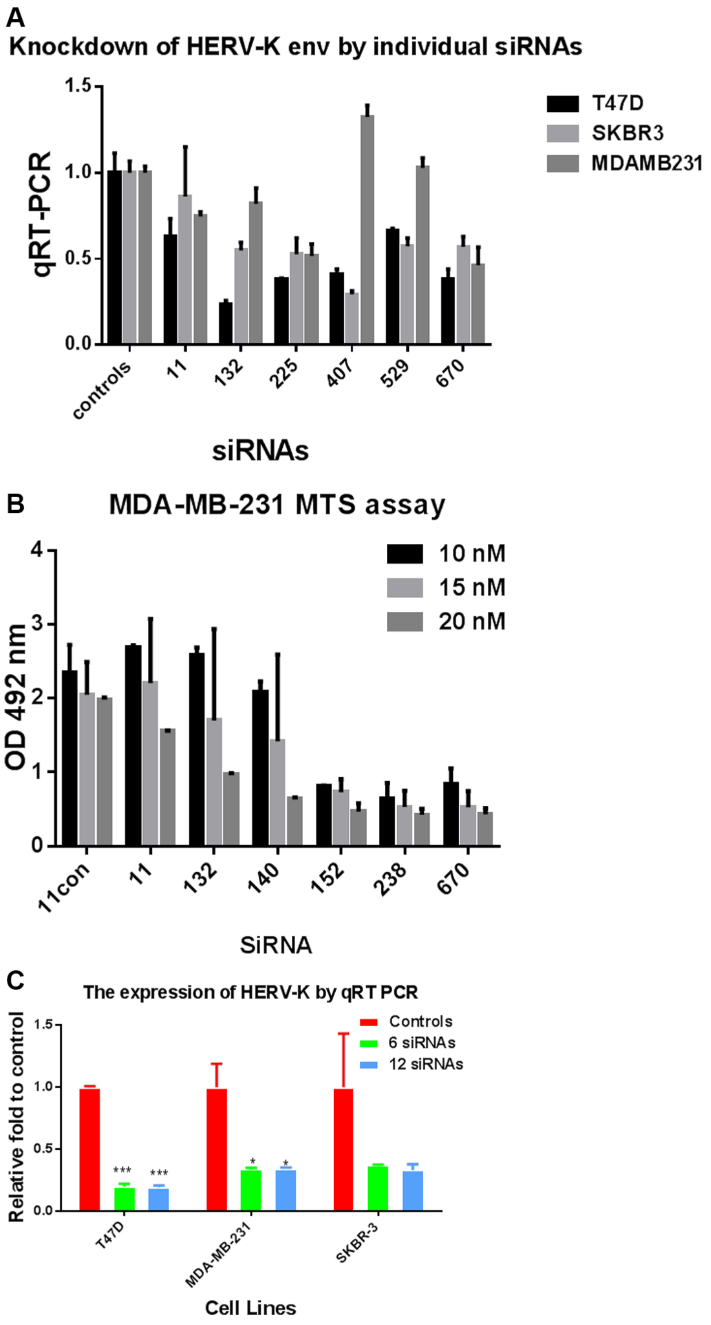

Supplementary Figure S1: Effects of siRNA treatment on expression of HERV-K in BC cells. (**A**) Six siRNAs were used to knock down the expression of HERV-K in three BC cell lines. (**B**) Cell proliferation was inhibited in MDAMB-231 cells treated with siRNAs that targeted HERV-K env (11, 132, and 670) or HERV-K Rec (140, 152, and 38) at various time points, assessed by 3-(4,5-dimethylthiazol-2-yl)-5-(3-carboxymethoxyphenyl)-2-(4-sulfophenyl)-2H-tetrazolium (MTS) assay, as we did previously [6]. (**C**) Significantly reduced expression of HERV-K was demonstrated in three BC cell lines treated with 6 siRNAs targeting HERV-K env or 12 siRNAs targeting both HERV-K env and HERV-K Rec.

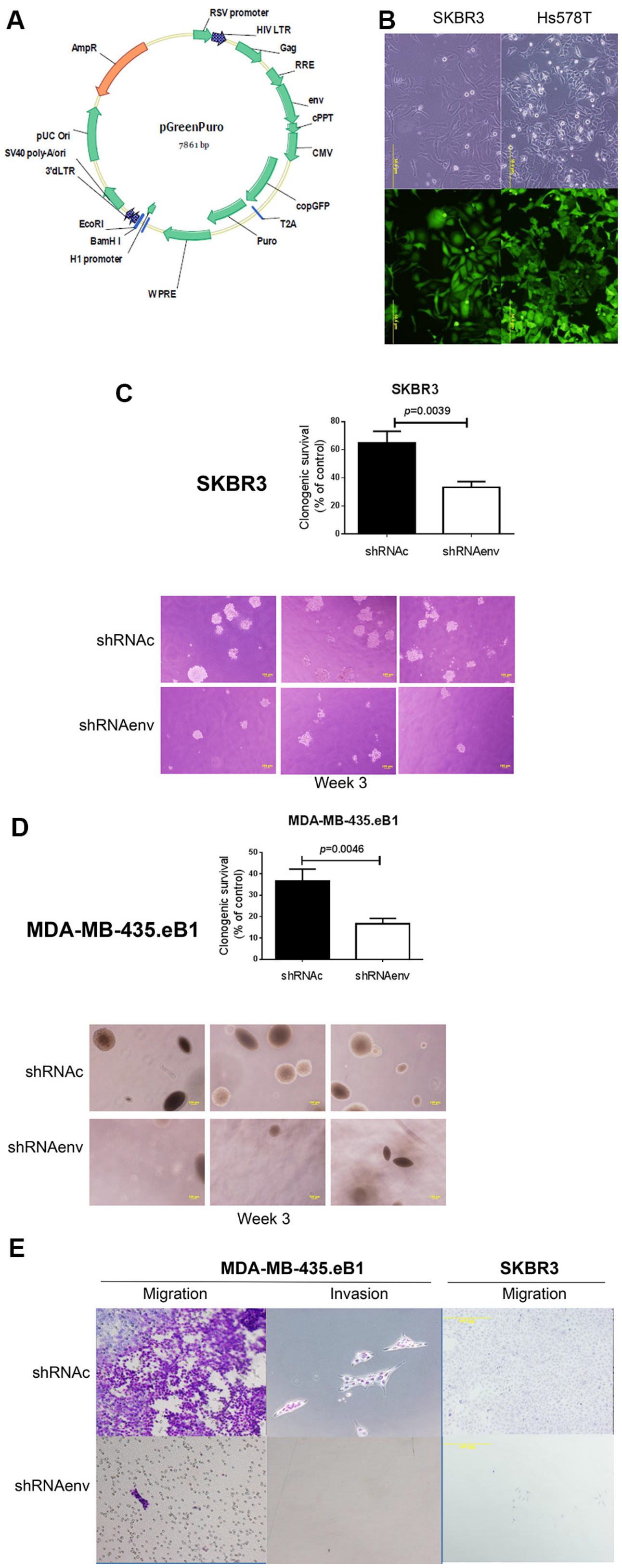

Supplementary Figure S2: Effects of HERV-K knockdown with an shRNA vector designed to express HERV-K shRNA or a scrambled control shRNA on colony growth, migration, and invasion of breast cancer cells. A shRNA sequence targeting the HERV-K env SU region (shRNAenv) and a matched scrambled shRNA sequence serving as a negative control (shRNAc) were cloned into the lentivector vector pGreenPuro at EcoRI and BamH I restriction enzyme sites (**A**). The transduction efficiency of shRNAenv in two cell lines is depicted (**B**). Reduced colony numbers in soft agarose were demonstrated for SKBR3 (**C**) ($n = 3$; $p = 0.0039$; 3 weeks post-seeding), and MDA-MB-435.eB1 (**D**) ($p = 0.0046$; 3 weeks post-seeding) cells. Decreased migration of MDA-MB-435.eB1 or SKBR3 cells and invasion of MDA-MB-435.eB1 stably transduced with shRNAenv was observed (**E**), compared with cells transduced with a scrambled shRNAc vector. The deviation (error bars) represent standard error of the mean (SEM), and the statistical test performed was unpaired t test.

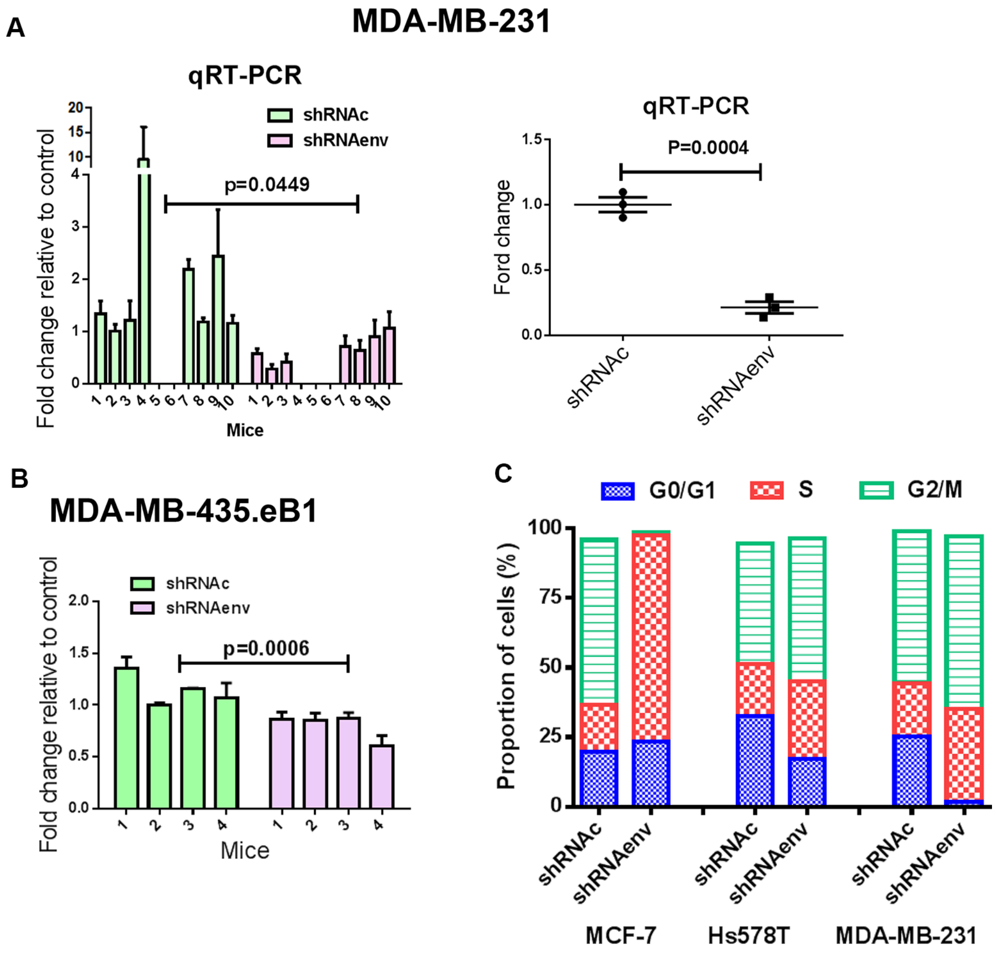

Supplementary Figure S3: Down-regulation of Kenv mRNA expression in xenograft tumor biopsies. Kenv mRNA expression was significantly decreased in the tumor biopsies of mice bearing cells transduced with shRNAenv compared with cells transduced with shRNAc, as assessed by qRT-PCR for MDA-MB-231 (**A**, left: n = 8 for shRNAc and n = 7 for shRNAenv; *p* = 0.0449; right: *n* = 3 for shRNAc and *n* = 4 for shRNAenv; *p* = 0.0004), from two experiments, and for MDA-MB-435.eB1 (**B**) (*p* = 0.0006). Cell cycle analysis was employed to determine the proportion of shRNAenv-transduced vs. shRNAc-transduced BC cells in each stage of the cell cycle. Percentages of G0/G1, S, and G_2-M phases in MCF-7, Hs578T, and MDA-MB-231 cells are shown (**C**). The deviation (error bars) represents standard error of the mean (SEM), and the statistical test performed was unpaired t test.

A

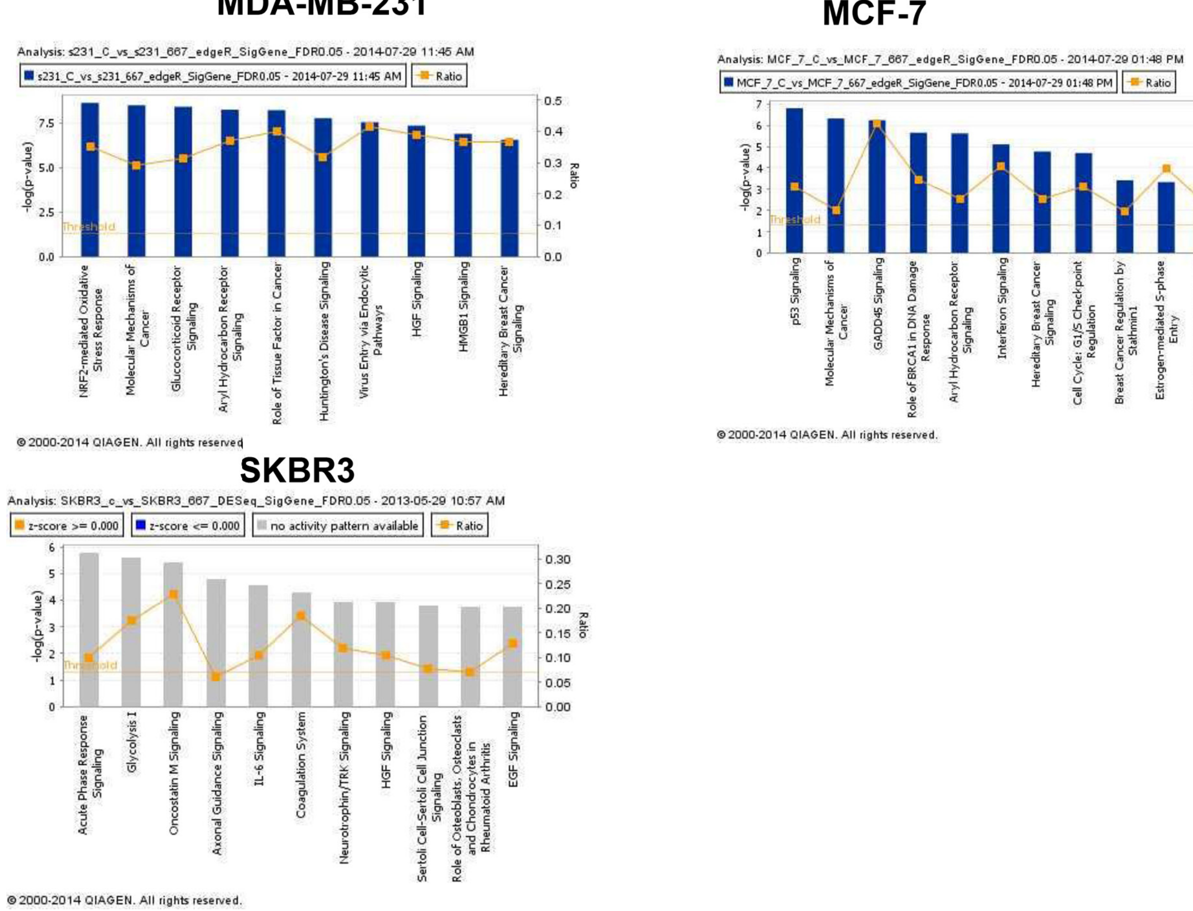
MDA-MB-231
Analysis: s231_C_vs_s231_667_edgeR_SigGene_FDR0.05 - 2014-07-29 11:45 AM
s231_C_vs_s231_667_edgeR_SigGene_FDR0.05 - 2014-07-29 11:45 AM
Ratio
-log(p-value)
Threshold
NRF2-mediated Oxidative Stress Response
Molecular Mechanisms of Cancer
Glucocorticoid Receptor Signaling
Aryl Hydrocarbon Receptor Signaling
Role of Tissue Factor in Cancer
Huntington's Disease Signaling
Virus Entry via Endocytic Pathways
HGF Signaling
HMGB1 Signaling
Hereditary Breast Cancer Signaling
© 2000-2014 QIAGEN. All rights reserved.
MCF-7
Analysis: MCF_7_C_vs_MCF_7_667_edgeR_SigGene_FDR0.05 - 2014-07-29 01:48 PM
MCF_7_C_vs_MCF_7_667_edgeR_SigGene_FDR0.05 - 2014-07-29 01:48 PM
Ratio
-log(p-value)
Threshold
p53 Signaling
Molecular Mechanisms of Cancer
GADD45 Signaling
Role of BRCA1 in DNA Damage Response
Aryl Hydrocarbon Receptor Signaling
Interferon Signaling
Hereditary Breast Cancer Signaling
Cell Cycle: G1/S Checkpoint Regulation
Breast Cancer Regulation by Stathmin1
Estrogen-mediated S-phase Entry
Pancreatic Adenocarcinoma Signaling
Granzyme A Signaling
© 2000-2014 QIAGEN. All rights reserved.
SKBR3
Analysis: SKBR3_c_vs_SKBR3_667_DESeq_SigGene_FDR0.05 - 2013-05-29 10:57 AM
z-score >= 0.000
z-score <= 0.000
no activity pattern available
Ratio
-log(p-value)
Threshold
Acute Phase Response Signaling
Glycolysis I
Oncostatin M Signaling
Axonal Guidance Signaling
IL-6 Signaling
Coagulation System
Neurotrophin/TRK Signaling
HGF Signaling
Sertoli Cell-Sertoli Cell Junction Signaling
Role of Osteoblasts, Osteoclasts and Chondrocytes in Rheumatoid Arthritis
EGF Signaling
© 2000-2014 QIAGEN. All rights reserved.

B

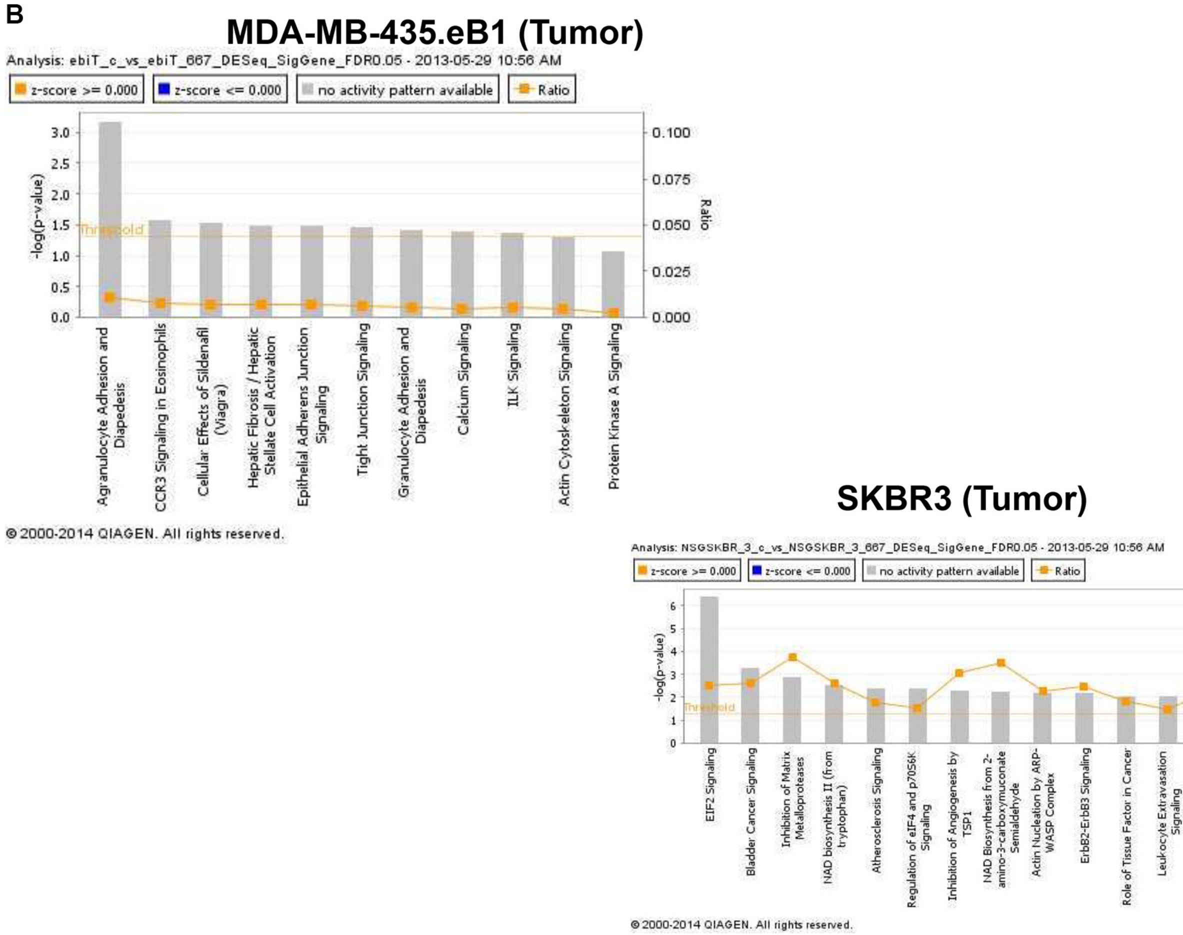
MDA-MB-435.eB1 (Tumor)
Analysis: ebiT_c_vs_ebiT_667_DESeq_SigGene_FDR0.05 - 2013-05-29 10:56 AM
z-score >= 0.000
z-score <= 0.000
no activity pattern available
Ratio
-log(p-value)
Threshold
Agranulocyte Adhesion and Diapedesis
CCR3 Signaling in Eosinophils
Cellular Effects of Sildenafil (Viagra)
Hepatic Fibrosis / Hepatic Stellate Cell Activation
Epithelial Adherens Junction Signaling
Tight Junction Signaling
Granulocyte Adhesion and Diapedesis
Calcium Signaling
ILK Signaling
Actin Cytoskeleton Signaling
Protein Kinase A Signaling
© 2000-2014 QIAGEN. All rights reserved.
SKBR3 (Tumor)
Analysis: NSGSKBR_3_c_vs_NSGSKBR_3_667_DESeq_SigGene_FDR0.05 - 2013-05-29 10:56 AM
z-score >= 0.000
z-score <= 0.000
no activity pattern available
Ratio
-log(p-value)
Threshold
EIF2 Signaling
Bladder Cancer Signaling
Inhibition of Matrix Metalloproteases
NAD biosynthesis II (from tryptophan)
Atherosclerosis Signaling
Regulation of eIF4 and p70S6K Signaling
Inhibition of Angiogenesis by TSP1
NAD Biosynthesis from 2-amino-3-carboxymuconate Semialdehyde
Actin Nucleation by ARP-WASP Complex
ErbB2-ErbB3 Signaling
Role of Tissue Factor in Cancer
Leukocyte Extravasation Signaling
Cell Cycle: G1/S Checkpoint Regulation
© 2000-2014 QIAGEN. All rights reserved.

C

MDA-MB-231

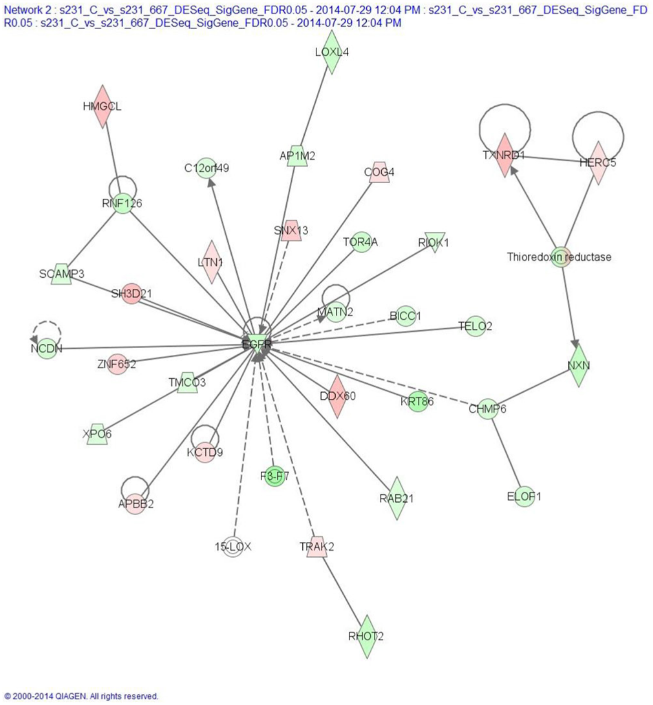

MCF-7

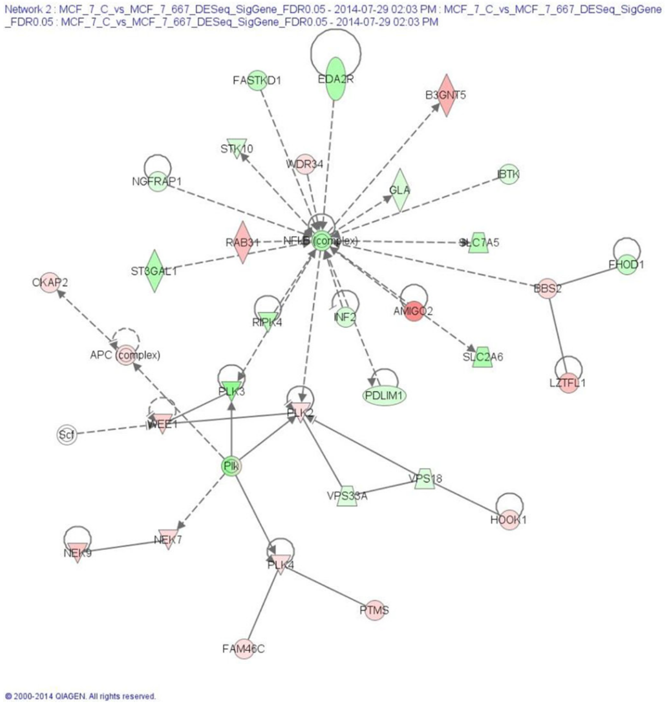

D

MDA-MB-435.eB1 (Tumor)

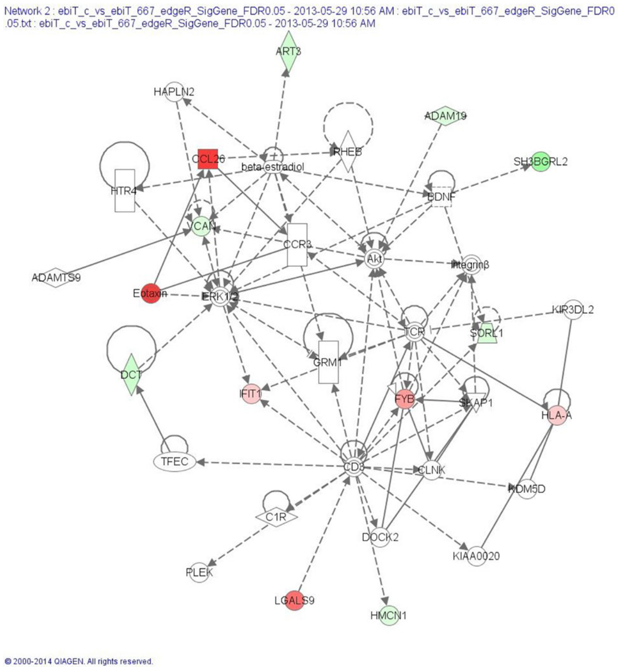

SKBR3 (Tumor)

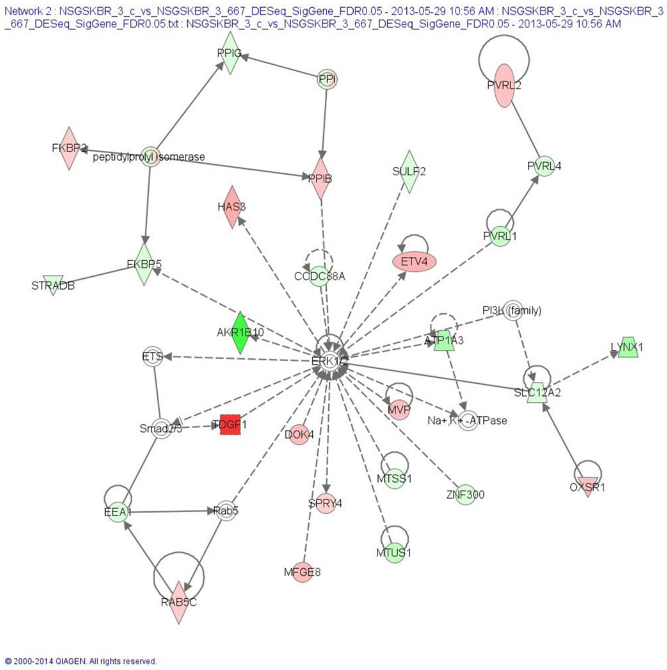

E

SKBR3 (Tumor) **SKBR3**

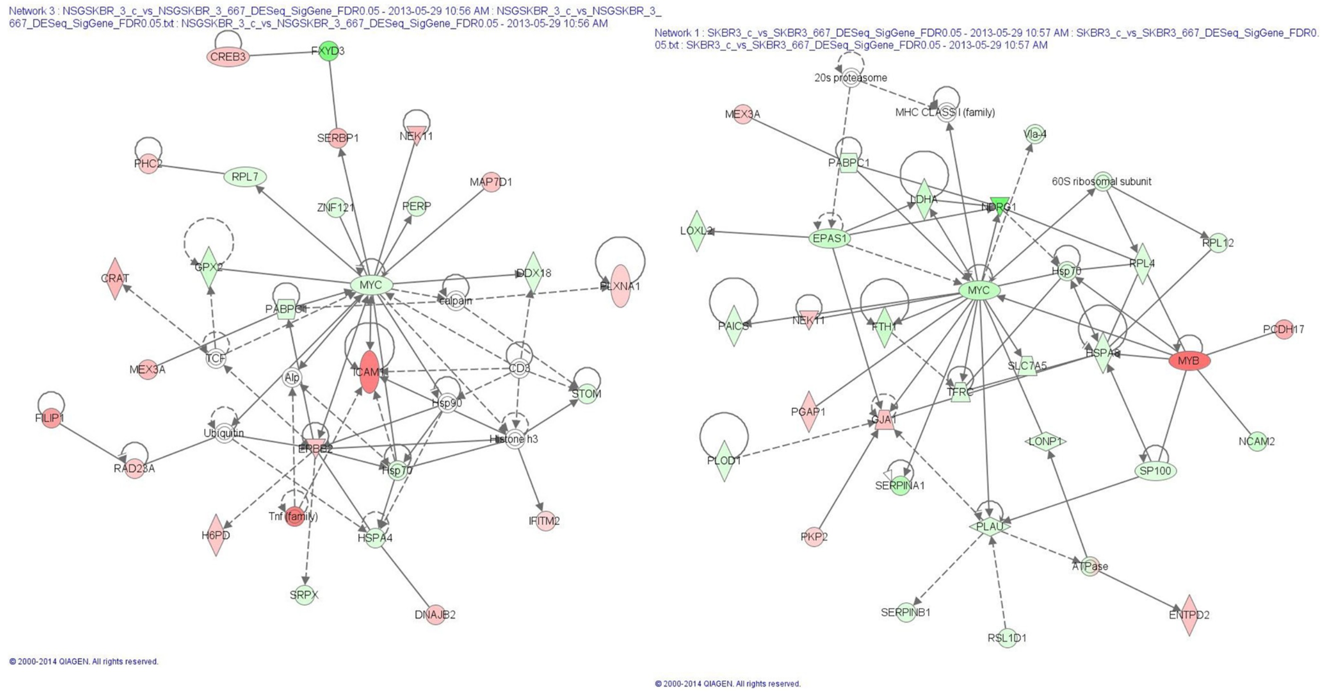

Supplementary Figure S4: Pathway analysis of RNA-Seq data from shRNAenv and shRNAc BC cells. Genes differentially expressed between shRNAenv-transduced and shRNAc-transduced BC cells or xenograft models, along with their expression levels, were imported to the Ingenuity Pathway Analysis (IPA) program to construct interacting pathways. (**A**) Canonical pathways determined for the BC cell lines MDA-MB-231, MCF-7, and SKBR3, and (**B**) for xenograft models MDA-MB-435.eB1 (Tumor), and SKBR3 (Tumor) are presented. All pathways showed significant differences between shRNAenv and shRNAc BC cells, with *p* values as indicated. (**C**) Network 2 was developed from BC cell lines MDA-MB-231 (EGFR, left panel) and MCF-7 (NFKβ, right panel), and (**D**) from tumors of xenograft mice bearing MDA-MB-435.eB1 (ERK1/2, left panel) and SKBR3 (ERK1/2, right panel) cells, by comparing shRNAenv and shRNAc RNA-Seq gene expression. Network 3 and 1 was developed from SKBR3 Tumor (MYC, left panel) or cells (MYC, right panel) by comparing shRNAenv-transduced and shRNAc-transduced RNA-Seq gene expression (**E**). The intensity of the node color indicates the degree of upregulation (red) or downregulation (green) in treated BC cell lines. Solid and dashed lines indicate direct and indirect interactions, respectively. Different shapes of the nodes, including square, circle, diamond, rectangles etc., represent functional classification of the genes.

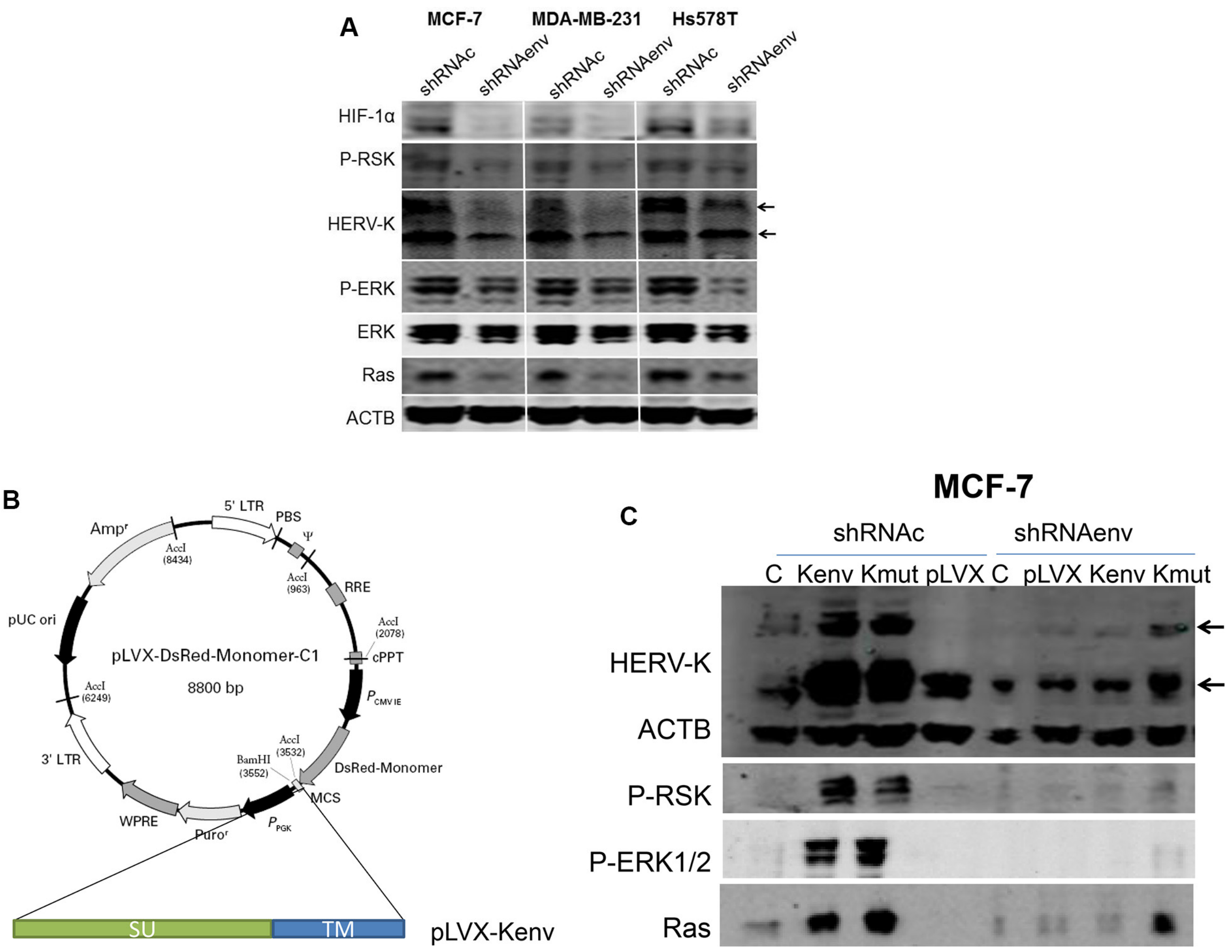

Supplementary Figure S5: Differential effects of downregulated and upregulated HERV-K expression. (**A**) Immunoblot analysis showed reduced expression of HIF-1a, p-RSK, p-ERK 1 or 2, and Ras in shRNAenv transduced MCF-7, MDA-MB-231, and Hs578T cells only when there was also shRNAenv-induced reduced expression of HERV-K Env protein in these cell lines. (**B**) HERV-K env sequence was cloned into lentivector vector pLVX-DsRed-Monomer-C1 (pLVX) at BsaI (insert)/EcoRI (vector) and XmaI restriction enzyme sites. (**C**) Immunoblot assays showed increased levels of HERV-K, p-RSK, p-ERK 1/2, and Ras protein in MCF-7 shRNAc cells transduced with Kenv or Kmut, and in MCF-7 shRNAenv cells transduced with Kmut.

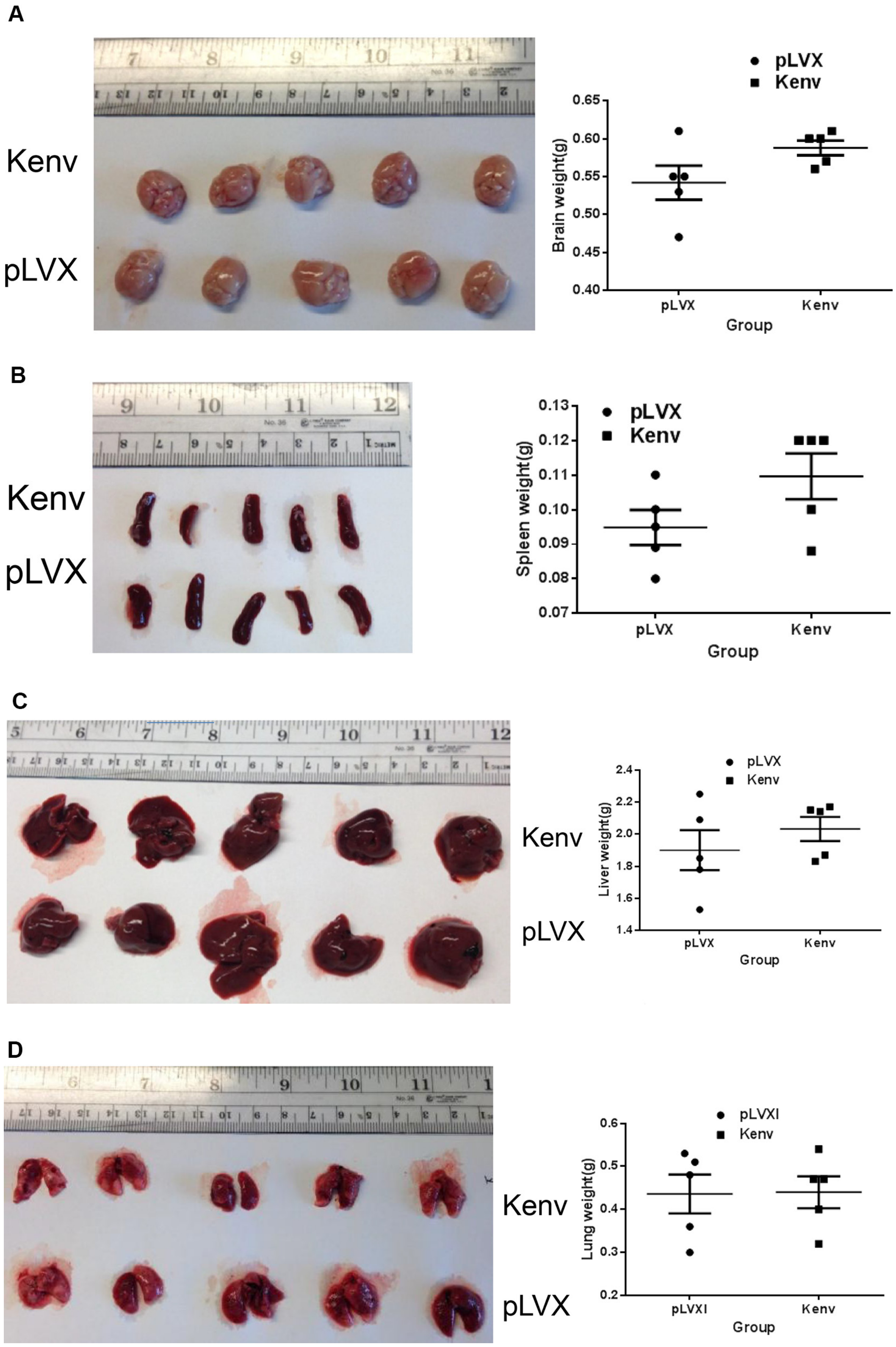
A
Kenv
pLVX
pLVX
Kenv
Brain weight(g)
0.65
0.60
0.55
0.50
0.45
0.40
pLVX
Kenv
Group
B
Kenv
pLVX
pLVX
Kenv
Spleen weight(g)
0.13
0.12
0.11
0.10
0.09
0.08
0.07
pLVX
Kenv
Group
C
Kenv
pLVX
pLVX
Kenv
Liver weight(g)
2.4
2.2
2.0
1.8
1.6
1.4
pLVX
Kenv
Group
D
Kenv
pLVX
pLVXI
Kenv
Lung weight(g)
0.6
0.5
0.4
0.3
0.2
pLVXI
Kenv
Group

E

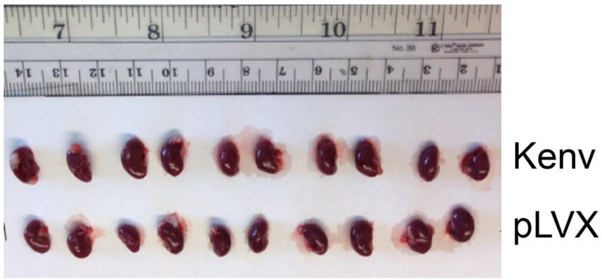

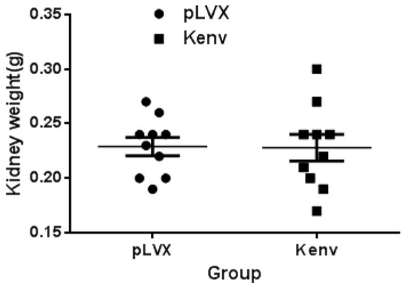

Supplementary Figure S6: Effect of HERV-K overexpression on metastasis in mouse xenografts. Tissue weights were compared in tumor bearing NSG mice (*n* = 5) bearing MDA-MB-231 cells transduced with Kenv or pLVX control plasmid, and sacrificed at 49 days post-injection. Slight but non-significant increases in brain (**A**) (p = 0.097), spleen (**B**) (p = 0.1143), and liver weights (**C**) (p = 0.391) were also demonstrated for xenografts bearing Kenv cells, compared with pLVX cells. No significant differences between the groups in lung (**D**) and kidney (**E**) weights were detected. The deviation (error bars) represents standard error of the mean (SEM), and the statistical test performed was unpaired *t* test.
